# The CspC pseudoprotease regulates germination of *Clostridioides difficile* spores in response to multiple environmental signals

**DOI:** 10.1371/journal.pgen.1008224

**Published:** 2019-07-05

**Authors:** Amy E. Rohlfing, Brian E. Eckenroth, Emily R. Forster, Yuzo Kevorkian, M. Lauren Donnelly, Hector Benito de la Puebla, Sylvie Doublié, Aimee Shen

**Affiliations:** 1 Department of Molecular Biology and Microbiology, Tufts University School of Medicine, Boston, Massachusetts, United States of America; 2 Department of Microbiology and Molecular Genetics, University of Vermont, Burlington, Vermont, United States of America; 3 Sackler School of Graduate Biomedical Sciences, Tufts University School of Medicine, Boston, Massachusetts, United States of America; The University of Texas Health Science Center at Houston, UNITED STATES

## Abstract

The gastrointestinal pathogen, *Clostridioides difficile*, initiates infection when its metabolically dormant spore form germinates in the mammalian gut. While most spore-forming bacteria use transmembrane germinant receptors to sense nutrient germinants, *C*. *difficile* is thought to use the soluble pseudoprotease, CspC, to detect bile acid germinants. To gain insight into CspC’s unique mechanism of action, we solved its crystal structure. Guided by this structure, we identified CspC mutations that confer either hypo- or hyper-sensitivity to bile acid germinant. Surprisingly, hyper-sensitive CspC variants exhibited bile acid-independent germination as well as increased sensitivity to amino acid and/or calcium co-germinants. Since mutations in specific residues altered CspC’s responsiveness to these different signals, CspC plays a critical role in regulating *C*. *difficile* spore germination in response to multiple environmental signals. Taken together, these studies implicate CspC as being intimately involved in the detection of distinct classes of co-germinants in addition to bile acids and thus raises the possibility that CspC functions as a signaling node rather than a ligand-binding receptor.

## Author summary

The major nosocomial pathogen *Clostridioides difficile* depends on spore germination to initiate infection. Interestingly, *C*. *difficile*’s germinant sensing mechanism differs markedly from other spore-forming bacteria, since it uses bile acids to induce germination and lacks the transmembrane germinant receptors conserved in almost all spore-forming organisms. Instead, *C*. *difficile* is thought to use CspC, a soluble pseudoprotease, to sense these unique bile acid germinants. To gain insight into how a pseudoprotease senses germinant and propagates this signal, we solved the crystal structure of *C*. *difficile* CspC. Guided by this structure, we identified mutations that alter the sensitivity of *C*. *difficile* spores to not only bile acid germinant but also to amino acid and calcium co-germinants. Taken together, our study implicates CspC in either directly or indirectly sensing these diverse small molecules and thus raises new questions regarding how *C*. *difficile* spores physically detect bile acid germinants and co-germinants.

## Introduction

*Clostridioides difficile*, previously classified as *Clostridium difficile*, is a Gram-positive, spore-forming, obligate anaerobe that is a leading cause of health-care associated infections and gastroenteritis-associated death worldwide [1]. In the United States (US) alone, *C*. *difficile* causes over 500,000 infections per year, leading to ~29,000 deaths and over $5 billion in medical costs [2]. While immunocompetent individuals are usually protected from *C*. *difficile* infection by the intestinal microflora, antibiotic treatment can render individuals susceptible to *C*. *difficile* infections due to disruption of the protective gut microbiota [3–5].

*C*. *difficile* infections are characterized by high rates of disease recurrence: approximately one in five patients that recover from a *C*. *difficile* infection will acquire a second infection within three months [2, 6, 7]. Both *C*. *difficile*’s vegetative cell and spore form contribute to recurrent infections [1, 8]: the vegetative form of *C*. *difficile* antagonizes growth of the protective microbiota by producing inflammation-inducing toxins [5], while its dormant spore form, which can resist commonly used disinfectants and harsh conditions [9, 10], allows *C*. *difficile* to outlast antibiotic treatment and persist in the environment for long periods of time [11].

Since the vegetative form of *C*. *difficile* cannot survive outside the anaerobic environment of the gastrointestinal tract, *C*. *difficile*’s aerotolerant, dormant spore form is its major infectious particle [9]. Consequently, *C*. *difficile* infections depend upon its spores germinating. When ingested *C*. *difficile* spores reach the small intestine, they sense mammalian-specific bile acids, which initiate a signaling cascade that allows spores to exit dormancy during germination [12–14]. Germinating spores outgrow to form the vegetative, toxin-producing cells that are responsible for *C*. *difficile* disease symptoms, which can range from severe diarrhea to pseudomembraneous colitis, toxic megacolon, and death [1, 6]. Since germination is required for *C*. *difficile* to initiate infection, therapeutics that inhibit germination to prevent *C*. *difficile* infection are currently being developed [15–18]. However, the molecular mechanisms underlying the *C*. *difficile* germination signaling cascade are poorly understood.

Indeed, recent studies indicate that *C*. *difficile*’s germination pathway differs significantly from other spore forming organisms [19, 20]. Almost all spore-forming organisms studied to date encode transmembrane germinant receptors of the Ger family [21, 22], which sense nutrient germinants, like amino acids, nucleic acids, and sugars [23]. In contrast, *C*. *difficile* does not encode Ger family receptors. Furthermore, the primary germinants for *C*. *difficile* are cholate-derived bile acids (especially taurocholate [24]), which are not known to be a nutrient source. Instead, genetic data suggest that *C*. *difficile* uses a soluble pseudoprotease, CspC, to directly sense bile acids [12]. CspC was identified in a genetic screen for mutants that germinate in response to the bile acid, chenodeoxycholate [12], which typically acts as a competitive inhibitor of germination [25]. Remarkably, a single point mutation in CspC (glycine 457 to arginine, G457R) expanded *C*. *difficile*’s germinant specificity to permit germination in response to chenodeoxycholate as well as taurocholate [12]. Since point mutations in CspC that prevent spore germination were also identified, CspC was proposed to be the bile acid germinant receptor [12].

Interestingly, *C*. *difficile* CspC is a catalytically inactive member of the Csp family of proteases, which were first identified in *Clostridium perfringens* as being responsible for proteolytically activating the cortex hydrolase, SleC [26, 27]. Active SleC degrades the thick protective cortex layer [28, 29], a step that is essential for all spores to exit dormancy [23]. *C*. *perfringens* strains encode one or more of three Csps, CspA, CspB, and CspC [30, 31], which likely have redundant functions during germination [31]. We previously solved the crystal structure of CspB from *C*. *perfringens* and confirmed that Csps are structurally similar to other subtilisin-like serine proteases [32, 33]. CspB protease activity depends on a catalytic triad consisting of Asp, His, and Ser, and the prodomain of CspB acts as an intramolecular chaperone that is auto-processed upon proper folding of CspB’s subtilisin-like serine protease domain [32]. However, unlike other subtilisin-like serine proteases studied to date, the prodomain of CspB stays associated with the subtilase domain after cleavage and sterically occludes CspB’s active site [32].

While C. *difficile* strains encode homologs of all three Csp proteins, only CspB has an intact Asp, His, and Ser catalytic triad, since CspA and CspC carry mutations in two of the catalytic residues that render them pseudoproteases [30]. As a result, only CspB can proteolytically activate SleC during *C*. *difficile* spore germination [32]. Furthermore, CspB is produced as a fusion to CspA in sporulating *C*. *difficile* cells that subsequently undergoes interdomain processing to release CspB and CspA as separate proteins, with the individual proteins being detected in mature spores [30, 32]. Notably, the CspBA fusion protein and the pseudoprotease nature of CspA and CspC appear to be unique to *C*. *difficile* and the Peptostreptococcaceae family [34], since members of the Clostridiaceae and Lachnospiraceae family exclusively produce Csp family proteases as individual proteins with intact catalytic triads [30]. In *C*. *difficile*, the CspA and CspC pseudoproteases both play critical and unique roles during spore germination. CspA controls CspC’s incorporation and/or stability in mature spores [30, 35], and as described earlier, CspC is the proposed bile acid germinant receptor [12].

To gain insight into the molecular mechanisms by which *C*. *difficile* spores sense germinant, we solved the crystal structure of *C*. *difficile* CspC to 1.55 Å resolution. This structure revealed unique features of the *C*. *difficile* CspC pseudoprotease when compared to the *C*. *perfringens* CspB protease. Structure-function analyses identified several flexible residues critical for CspC function that confer either hyper- or hypo-sensitivity to bile acid germinant. Further analyses revealed that some of these mutations alter sensitivity to amino acid and/or calcium co-germinants, which potentiate bile acid-induced germination in *C*. *difficile*. Since the mechanism by which co-germinants are sensed by *C*. *difficile* spores was previously unknown [19, 20], our study reveals for the first time, to our knowledge, that *C*. *difficile* CspC integrates multiple germinant and co-germinant signals to induce spore germination and raises important new questions regarding how these signals are specifically sensed and transduced.

## Results

### Overall structure of CspC

**Fig 1:**
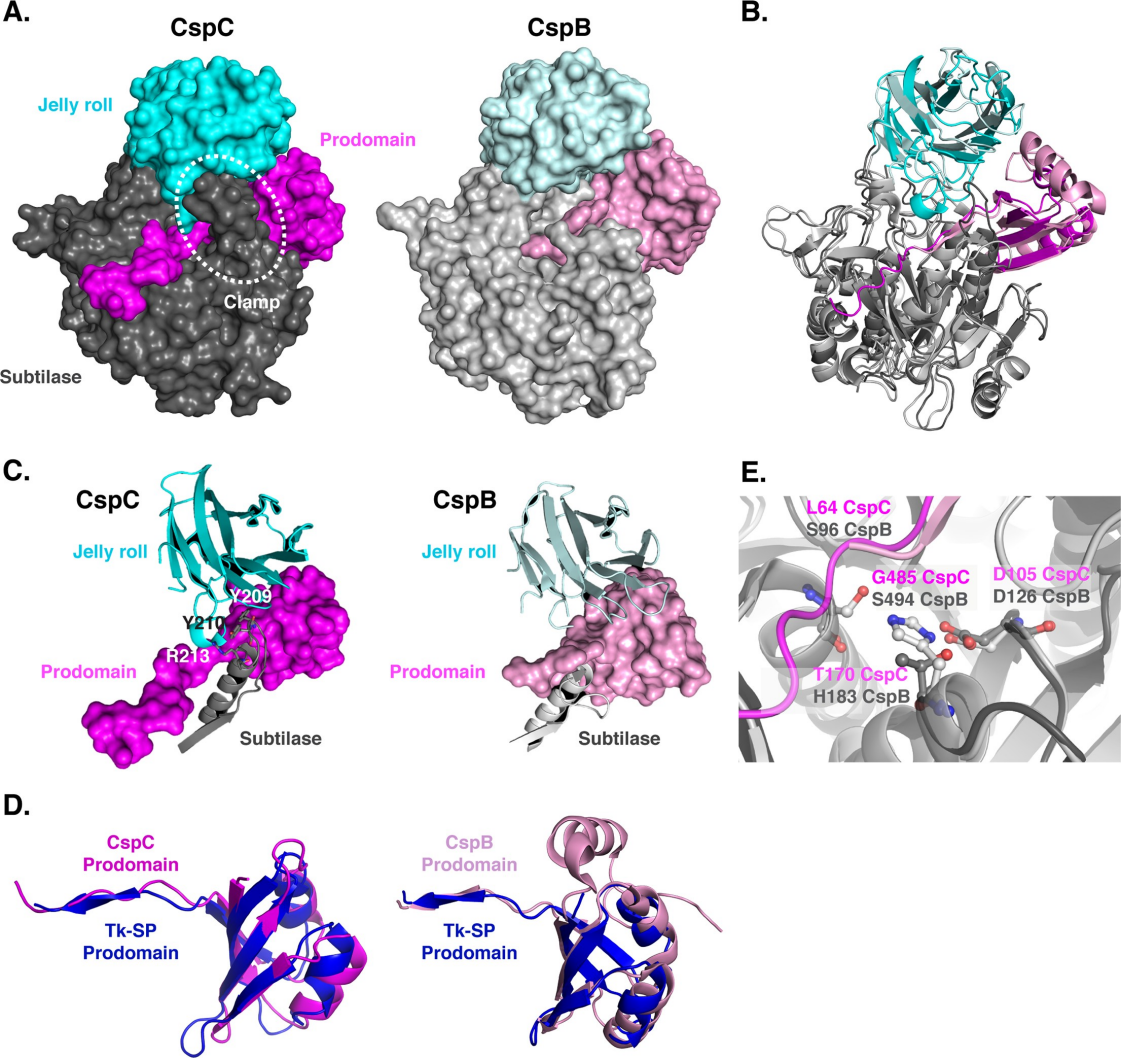
Structural comparison of *C*. *difficile* CspC to *C*. *perfringens* CspB. (A) Space-fill models of CspC and CspB [32] with the respective prodomains colored in pink, subtilase domains in grey, and jell roll domains in cyan (lighter tones denote CspB). The prodomain “clamp” in CspC formed by interactions between the jelly roll domain and subtilase domains is highlighted. (B) Ribbon representation overlay of CspC and CspB structures. A least squares superposition of the subtilase domain reveals a 1.03 Å rms over 1200 atoms of the backbone trace. (C) Comparison of the CspC prodomain “clamp” relative to CspB. The jelly roll domain interacts with a clamping loop from the CspC subtilase domain through which the CspC prodomain is threaded. Specific residues involved in the CspC jelly roll-subtilase domain interactions are marked. This interaction is absent in CspB due to a shorter subtilase domain loop, which is in a different orientation, and the disorder of the jelly roll domain interacting loop. (D) Superposition of the CspC and CspB prodomains, respectively, with the canonical prodomain from Tk-subtilisin [36] (blue). (E) Overlay of the CspB catalytic triad (S494, H183 and D126) to CspC (G485, T170 and D105). The position of the terminal residue of the CspB prodomain (S96) as well as the equivalent residue in CspC’s uncleaved prodomain (L64) is indicated.

To determine how *C*. *difficile* CspC directly senses bile acid germinants, we attempted to crystallize recombinant C-terminally His_6_-tagged CspC (both the wild-type and the G457R variant, the latter of which was previously shown to expand the germinant specificity of *C*. *difficile* spores to include chenodeoxycholate [12]) alone or in the presence of either taurocholate or chenodeoxycholate. While crystals were observed in all three conditions using wild-type CspC, no crystals were obtained in our screening with the G457R variant. Furthermore, diffraction quality crystals were only obtained with wild-type CspC in the absence of bile acids. Determination of the crystal structure of the *C*. *difficile* CspC pseudoprotease revealed that it shares a conserved three domain architecture with the *C*. *perfringens* CspB protease (Fig 1A), consisting of an N-terminal prodomain, subtilase domain, and jelly roll domain (Fig 1A, [32]). The jelly roll is a ~130 amino acid insertion in the subtilase domain that forms a β-barrel domain that appears unique to the Csp family of subtilisin like-serine proteases. This jelly roll domain provides CspB with remarkable structural rigidity, conferring thermostability and resistance to proteolysis [32]. In both CspB and CspC, the jelly roll domain emerges from the subtilase domain and extensively interacts with both the subtilase domain and the prodomain. Overall, the secondary structure of these proteins is highly similar (Fig 1B), with a 1.03 Å rmsd over 1200 atoms of the backbone trace.

Despite this shared secondary structure, *C*. *difficile* CspC does not autoprocess its prodomain due to substitutions in its catalytic triad. In addition, CspC’s prodomain is more closely associated with the subtilase and jelly roll domains due to a clamping loop shown in the surface representation (Fig 1A). This loop resides between beta strand 4 and helix 4 within the subtilase domain (residues 203–216) and interacts with the jelly roll domain via packing of tyrosine 209 and tyrosine 210 as well as hydrogen bonds from arginine 213 to backbone carbonyls in the 402–407 residue loop of the jelly roll domain (Fig 1C). In CspB, the equivalent subtilase domain loop is shorter (residues 214–225) and in a different conformation, and the jelly roll domain loop is disordered, possibly due to the autoprocessing of the prodomain by CspB’s subtilase domain (Fig 1C).

The prodomain of CspC aligns more closely with the canonical Tk-SP subtilisin prodomain [36] than to CspB because CspB’s prodomain is larger due to a helical insert (Fig 1D). Nevertheless, both prodomains share a similar orientation relative to the active (or degenerate) site residues (Fig 1E). As a result, leucine 64 of CspC (Fig 1E) is equivalent to the autocleavage site of the CspB *perfringens* prodomain, serine 96 [27, 32]), and would be positioned to undergo autoprocessing if CspC’s catalytic triad were intact. Interestingly, the degenerate site residues of CspC’s pseudotriad share the same orientation as the catalytic triad of CspB and other subtilisin-like serine proteases (Fig 1E).

### Structural flexibility of CspC appears to be important for function

**Fig 2:**
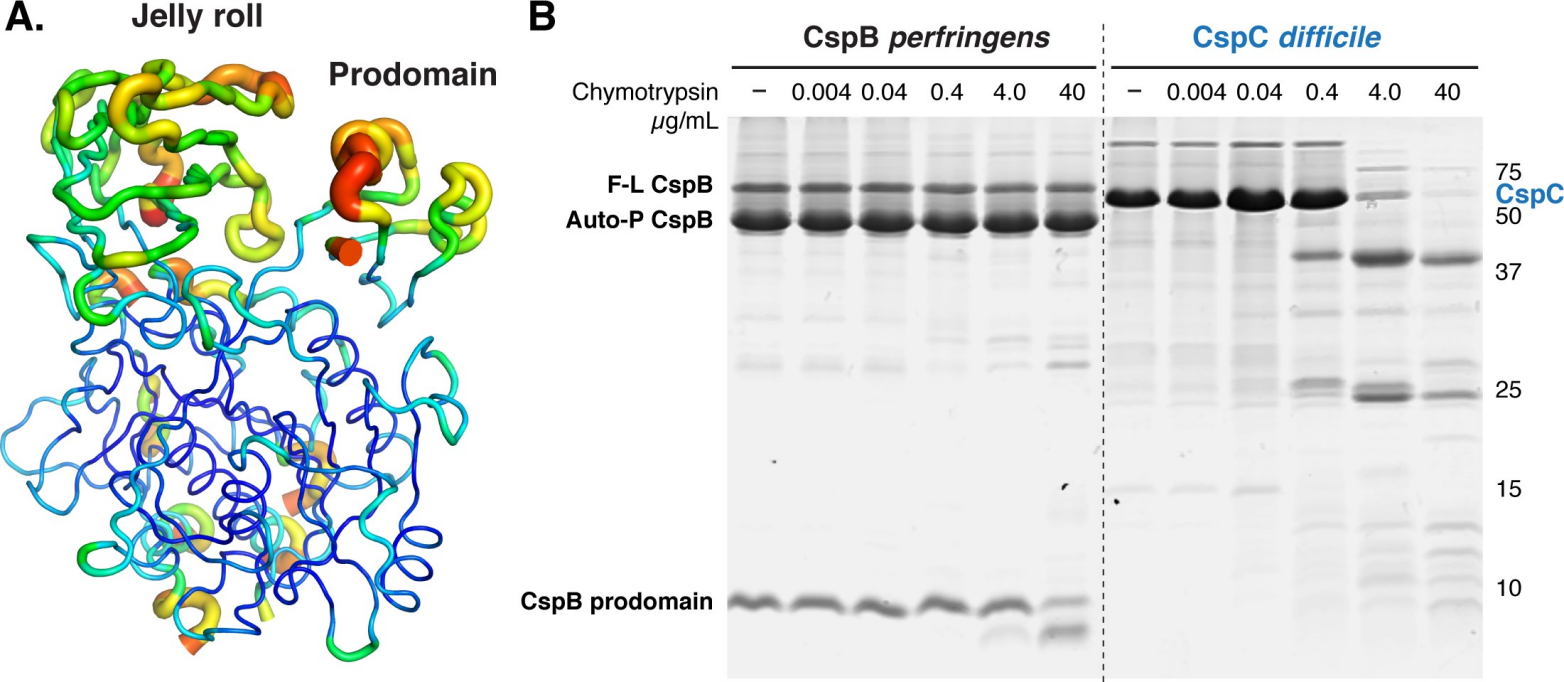
CspC is more conformationally flexible than CspB. (A) B-factor representation suggesting that the jelly roll and prodomains of CspC exhibit dynamic mobility. The higher relative B-factors are more striking for the jelly roll because there are strong crystal packing interfaces at two of the crystallographic two-folds. B-factor is indicated by color and line thickness. High B-factors represented by warm colors and a thick line; low B-factors represented by cool colors and a thin line. (B) Limited proteolysis of CspB and CspC. 15 μM of the proteins were incubated with increasing concentrations of chymotrypsin for 1 hr at 37˚C. F-L refers to full-length CspB, and Auto-P refers to CspB that has undergone autoprocessing to release the CspB prodomain.

Despite these similarities, CspC appeared to bind its prodomain more tightly than CspB. When the interfaces between each of the three subdomains were evaluated by buried surface area analysis, a larger contact area is observed between each of the domains in CspC as compared to CspB (S1 Table, 3200 Å^2^ for CspC vs. 2270 Å^2^ for CspB, calculated with PDBe PISA[37]). Surprisingly, even with the extensive interactions between the different sub-domains of CspC, residues in both the jelly roll domain and prodomain have higher B-factors than nearby residues in the subtilase domain (Fig 2A). Since B-factors measure the movement of a residue around its average position, *C*. *difficile* CspC would appear to have greater flexibility in the jelly roll domain and prodomain than in the subtilase domain. In contrast, the previously solved crystal structure of *C*. *perfringens* CspB showed little flexibility in terms of its B-factors and was highly resistant to proteolysis [32]. CspC’s flexibility was particularly unexpected given that the free energy of binding of the prodomain to the rest of the protein was -25.2 ΔG kcal/mol relative to -20.0 ΔG kcal/mol for CspB (S1 Table). To determine if the high B-factors in *C*. *difficile* CspC result in high conformational flexibility relative to *C*. *perfringens* CspB, we subjected purified *C*. *difficile* CspC and *C*. *perfringens* CspB to limited proteolysis. Consistent with prior work, purified *C*. *perfringens* CspB was highly resistant to proteolysis by chymotrypsin, whereas purified *C*. *difficile* CspC was considerably more sensitive **(**Fig 2B). These results indicate that *C*. *difficile* CspC is more conformationally dynamic than *C*. *perfringens* CspB under the conditions used for crystallization and limited proteolysis, although within the *C*. *difficile* spore, these proteins likely experience different environmental conditions and possibly different relative mobilities.

**Fig 3:**
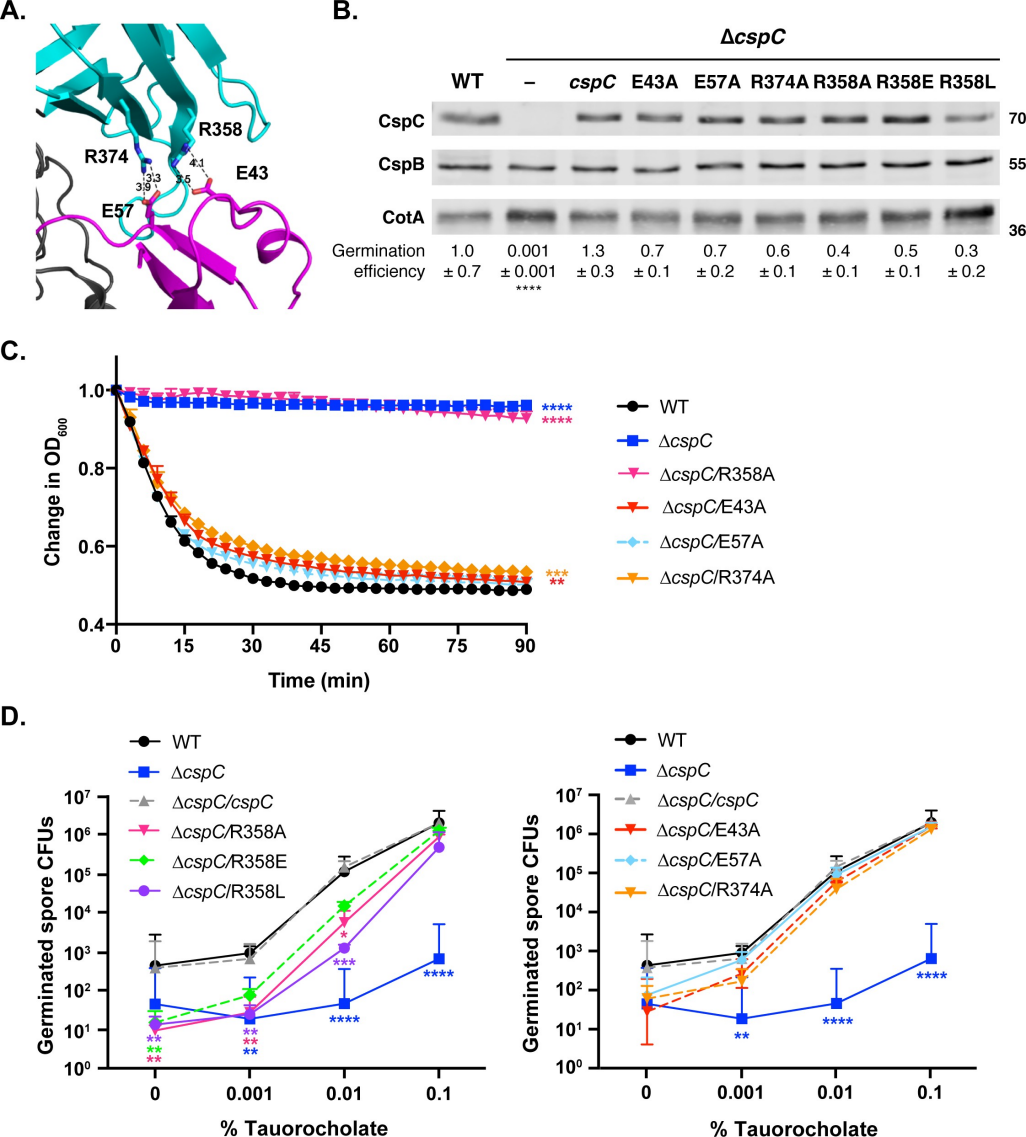
Mutations in the flexible residue, Arg358, decrease sensitivity to bile acid germinant. (A) Close-up view of dual salt-bridge interactions at the jellyroll domain-prodomain interface. The jelly roll domain is shown in cyan, and the prodomain is shown in magenta. The distances between the E57:R374 and R358:E43 salt bridge interactions are marked. (B) Western blot analyses of CspC and CspB levels in salt bridge mutant spores. CotA serves as a loading control. The results are representative of three biological replicates performed on three independent spore preps. The germination efficiency of the salt bridge mutants plated on BHIS media containing 0.1% taurocholate is also shown relative to wild type. No significant differences in germination were observed for the salt bridge mutants using a one-way ANOVA and Tukey’s test. **** p < 0.0001. (C) Optical density (OD_600_) analyses of spore germination over time in salt bridge mutants. Purified spores from the indicated strains were incubated in BHIS with and without 1% taurocholate. The change in OD_600_ represents the ratio of the OD_600_ of the treated to the untreated sample relative to the ratio at time zero. The results presented are representative of analyses of three biological replicates. The error bars indicate the standard deviation for each timepoint measured in three technical replicates. Lower error bars have been omitted to improve readability. (D) Germinant sensitivity of salt bridge mutant spores plated on BHIS containing increasing concentrations of taurocholate. The number of colony forming units (CFUs) that arose from germinating spores is shown in two graphs to improve readability. For all germination assays shown, the mean and standard deviations shown are based on three biological replicates performed on three independent spore purifications. Lower error bars have been omitted to improve readability. Statistical significance relative to wild type was determined using a two-way ANOVA and Tukey’s test. **** p < 0.0001, *** p < 0.001, ** p < 0.01, * p < 0.05.

To assess whether the relative mobility of *C*. *difficile* CspC is important for its function, we targeted residues exhibiting conformational flexibility in the structure for mutagenesis. Both arginine 358 in the jelly roll and glutamate 43 in the prodomain have higher average B-factors (49.1 and 55.8 Å^2^, respectively, vs. an average of 27.2 Å^2^). These two residues form a salt bridge adjacent to another salt bridge between arginine 374 in the jelly roll and glutamate 57 in the prodomain, which have B-factors of 43.9 and 33.6 Å^2^, respectively (Fig 3A). To test if the flexible Arg358 and Glu43 residues, or the neighboring salt bridge residues, are important for CspC function, we generated strains producing alanine substitutions of Glu43, Glu57, Arg374, and Arg358 and determined the impact of these mutations on spore germination efficiency. Purified spores were plated on BHIS media containing 0.1% taurocholate germinant, and the number of colony forming units (CFUs) that arose from germinating spores relative to wild type and the wild-type *cspC* complementation strain were determined. Similar to the phenotypes of other germination-receptor mutants and our own previously published results [35, 38–40], Δ*cspC* spores exhibited low levels of “spontaneous” germination when plated on BHIS media alone. However, none of the salt bridge mutants exhibited statistically significant germination defects when plated on rich media containing 0.1% taurocholate (Fig 3B), although germination was slightly lower (2.5-fold) in the R358A mutant.

Interestingly, when the salt bridge mutants were tested in an optical density-based assay for spore germination, the R358A variant exhibited a severe defect in spore germination compared to wild type spores during the 90 min assay length (p < 0.0001, Fig 3C). In contrast, the other salt bridge mutants tested exhibited germination responses similar to wild type, albeit slightly slower for the E43A and R347A mutant spores (p < 0.01). The optical assay measures the decrease in optical density of a population of germinating spores exposed to 1% taurocholate in rich media over time as the cortex is hydrolyzed and the core hydrates [41]. Since the time scale for the optical density assay was much shorter than the CFU-based germination assay, during which spores are constantly exposed to 0.1% taurocholate on rich media, we speculated that the R358A mutant might be less sensitive to germinant than wild type spores.

To test this possibility, we plated the salt bridge mutant spores on rich media containing increasing amounts of germinant (from 0 to 0.1%). Notably, the R358A mutants exhibited ~25-fold reduced sensitivity to germinants relative to the wild type when plated on media containing lower concentrations of germinant (i.e. 0.001% and 0.01% taurocholate, p < 0.01), whereas the other salt bridge mutants, E43A, E57A, and R374A, exhibited wild-type germinant sensitivity (Fig 3D).

We next assessed whether additional substitutions of Arg358 would affect the ability of *C*. *difficile* spores to sense germinant by generating strains that produced CspC with a negatively charged residue at residue 358, R358E, or a larger neutral residue at residue 358, R358L. The R358E substitution resulted in ~10-fold sensitivity to taurocholate at 0.001% and 0.01% relative to wild type, although this difference was not statistically significant, while the R358L mutation resulted in 30-100-fold decreases in germinant sensitivity at these concentrations relative to wild type.

Since none of the substitutions at Glu43, Glu57, Arg358, or Arg374 affected CspC levels in purified spores (Fig 3B), the Arg358 substitutions would appear to specifically disrupt CspC function and reduce the sensitivity of *C*. *difficile* spores to taurocholate germinant. Nevertheless, since mutation of Arg358’s salt bridge partner Glu43 (or the neighboring salt bridge) did not affect CspC function, the ability to Arg358 to bind Glu43 does not appear to affect CspC function. Instead, the physical properties of Arg358, including potentially its conformational flexibility, would appear to be critical for CspC signaling.

### Mutation of the flexible residues, G457 and R456, permits taurocholate-independent germination

**Fig 4:**
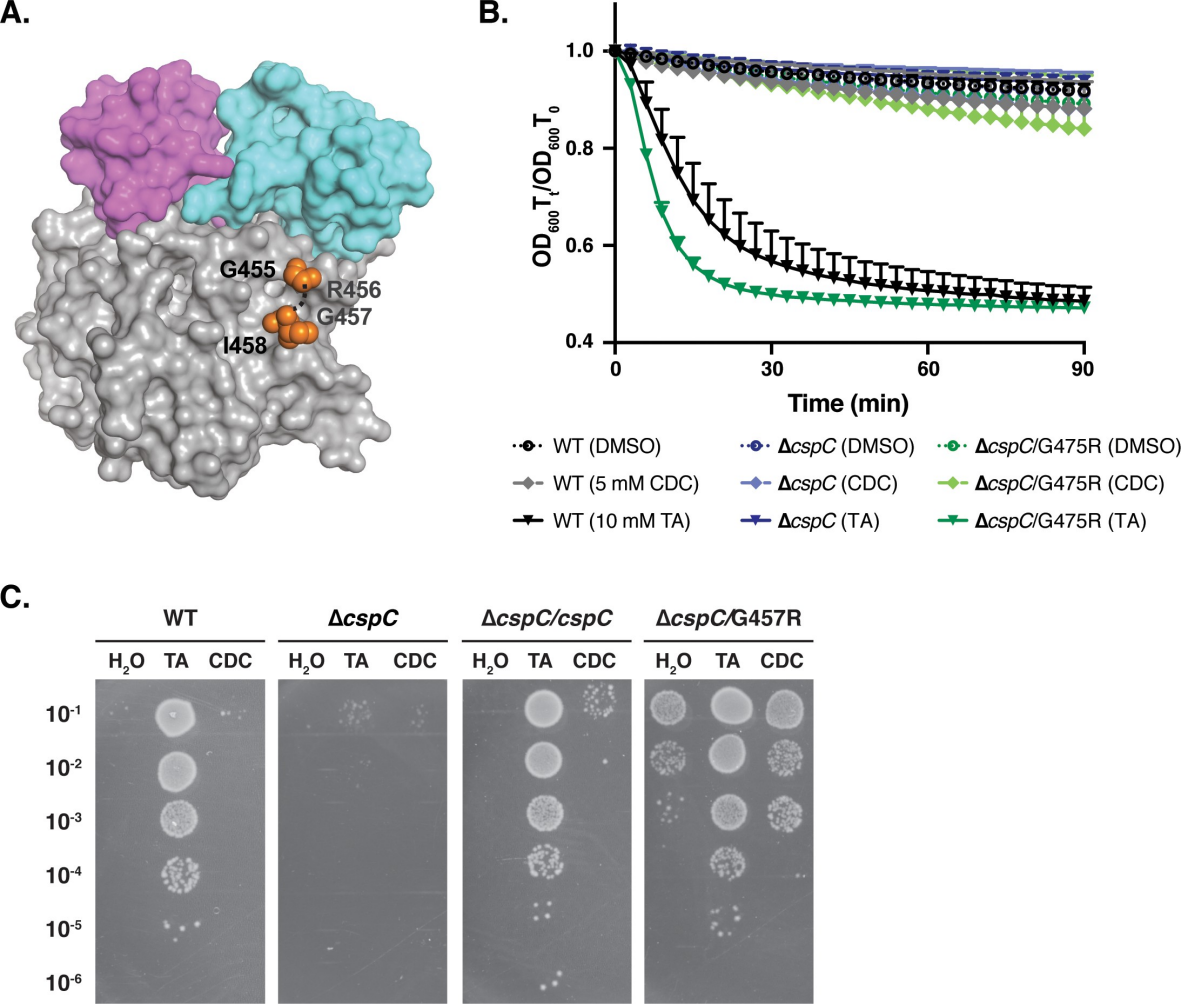
A G457R mutation results in taurocholate- and chenodeoxycholate-independent spore germination. (A) Space fill model of CspC highlighting the Gly445 and Ile458 residues flanking (orange) the solvent-exposed Arg456 and Gly457 flexible loop. This flexible loop is shown with a dotted line. (B) Optical density (OD_600_) analyses of spore germination over time in the indicated strains. Purified spores from the indicated strains were incubated in BHIS in the presence of either DMSO carrier, 10 mM taurocholate (~0.5%), or 5 mM chenodeoxycholate (~0.2%). The OD_600_ of the samples was monitored in a 96-well plate using a plate reader. The change in OD_600_ represents the OD_600_ of the sample at a given timepoint relative to its starting OD_600_ at time zero. The averages of the results from three biological replicates (i.e. three independent spore preparations) are shown. The error bars indicate the standard deviation for each timepoint measured. Lower error bars have been omitted to improve readability. No significant difference in spore germination was observed between the different strains treated with water or CDC. Only TA treatment resulted in statistically significant differences between Δ*cspC* and wild type (**** p < 0.0001). (C) Purified spores from wild type, Δ*cspC*, and Δ*cspC* complemented with either wild-type CspC or a G457R mutant were incubated in BHIS supplemented with either water, 1% TA (19 mM) taurocholate (TA), or 0.5% (12 mM) chenodeoxycholate (CDC) for 30 minutes at 37˚C then serially diluted in PBS and plated onto BHIS media lacking germinant. Colonies formed after a 24 hr incubation at 37˚C are shown.

Two additional residues exhibited considerable conformational flexibility in the CspC structure: arginine 456 and glycine 457, which lacked sufficient electron density to be included in our model for CspC (Fig 4A). As described earlier, Gly457 had previously been shown by Francis *et al*. to affect the germinant specificity of *C*. *difficile* spores, with a G457R substitution enabling *C*. *difficile* spores to germinate in response to chenodeoxycholate [12], which typically inhibits *C*. *difficile* spore germination [25]. This observation led to the hypothesis that CspC directly recognizes bile acids using glycine 457 [12].

Interestingly, Gly457 and Arg456 likely sit at the top of a pocket formed on the surface of the subtilase domain that could potentially accommodate a molecule of taurocholate. To test the importance of these residues, we individually mutated Gly457 to arginine (G457R) and Arg456 to Gly (R456G, Fig 4A). These mutations generate either two consecutive arginines or two consecutive glycines in the unstructured region, respectively. Before characterizing the germinant sensitivity and specificity of these strains, we first tested if the G457R mutation in the 630Δ*erm* strain background would permit germination in response to chenodeoxycholate as previously described for a G457R mutant in the UK1 strain background [12]. To this end, we monitored the change in optical density of germinating spores in response to taurocholate and chenodeoxycholate as previously described [12]. In our study, the *cspC*_G457R_ allele was expressed from the 630Δ*erm* chromosome, while the previous work expressed this mutant allele from a multicopy plasmid in strain UK1. CspC_G457R_ mutant spores germinated faster than wild-type spores in response to 10 mM taurocholate (~0.5%), suggesting that the glycine to arginine substitution at residue 457 increases the responsiveness of spores to taurocholate (Fig 4B). In contrast with the prior report [12], CspC_G457R_ spores exposed to 5 mM (~0.5%) chenodeoxycholate failed to decrease in optical density over time (Fig 4B). Furthermore, no evidence of spore germination was observed by phase-contrast microscopy, since CspC_G457R_ and control wild-type, Δ*cspC*, and Δ*cspC*/*cspC* spores remained phase-bright during a 20 min exposure to chenodeoxycholate, whereas the majority of CspC_G457R_, wild-type, and Δ*cspC/cspC* spores germinated and became phase-dark, indicative of core rehydration, upon exposure to taurocholate (S1A Fig).

We suspect that the decrease in optical density of CspC_G457R_ mutant spores reported by Francis *et al*. is caused by chenodeoxycholate forming a precipitate in the presence of *C*. *difficile* spores. We could recapitulate the optical density drop for CspC_G457R_ mutant spores when chenodeoxycholate was re-suspended in an older stock of DMSO; this DMSO stock resulted in a precipitate collecting at the bottom of the wells (S1B Fig). However, since this optical density decrease was also observed for the negative control, Δ*cspC* spores, as well as wild-type and Δ*cspC*/*cspC* spores, chenodeoxycholate can cause non-specific effects on optical density depending on the assay conditions.

To avoid these experimental artifacts, we assessed the ability of CspC_G457R_ spores to germinate in response to pre-treatment with either taurocholate or chenodeoxycholate and form colonies when plated on media lacking germinant (BHIS). Colonies that form on BHIS alone arise from spores that germinated during the transient pre-treatment with bile acids. Wild-type and wild-type complementation spores formed colonies only after pre-treatment with taurocholate and not chenodeoxycholate (Fig 4C and S2 Fig). CspC_G457R_ mutant spores formed colonies in response to pre-treatment with both taurocholate and chenodeoxycholate, albeit ~100-fold less efficiently with chenodeoxycholate. However, CspC_G457R_ mutant spores formed similar number of colonies with no bile acid pretreatment (water pretreatment) as with chenodeoxycholate pretreatment, indicating that these spores are germinating independent of the bile acid pretreatment.

Since the rich BHIS media used in the experiments detailed above is poorly defined, we tested whether CspC_G457R_ spores would also germinate on a more minimal medium (*C*. *difficile* defined-media, CDDM, [42]). Pre-treatment of wild-type, Δ*cspC*, Δ*cspC*/*cspC*, and Δ*cspC*/G457R mutant spores with either water, taurocholate, or chenodeoxycholate followed by plating on CDDM alone yielded results nearly identical to that obtained on BHIS alone (S2 Fig). Thus, CspC_G457R_ mutant spores can germinate independently of a bile acid signal and appear to sense something present in both undefined rich media (BHIS) and minimal media (CDDM). It should be noted that these mutant spores nevertheless still respond to taurocholate as a germinant, since CFUs increase by ~2-logs upon taurocholate pre-treatment (Fig 4 and S2 Fig).

**Fig 5:**
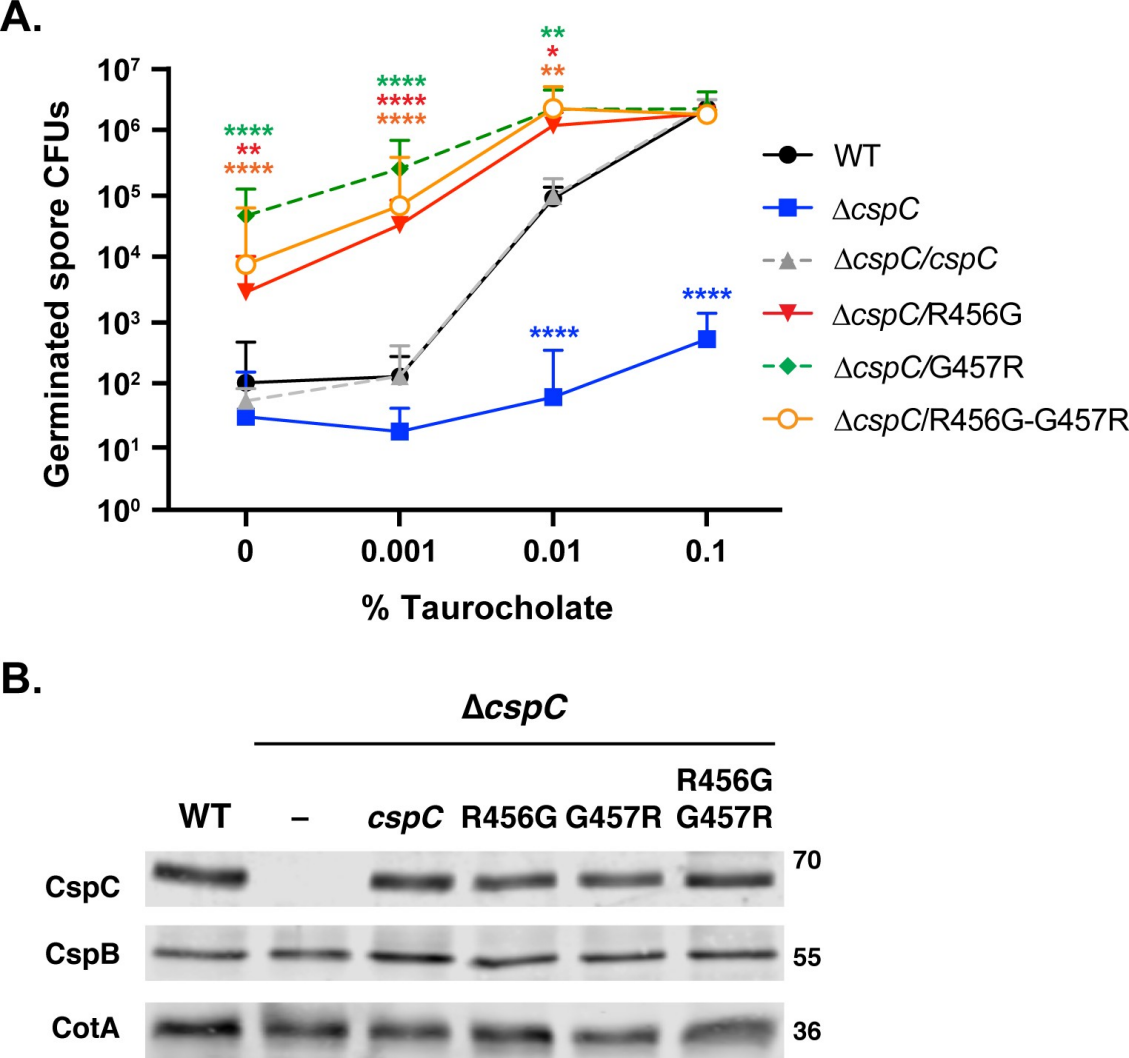
Mutations in the unstructured Arg456 and Gly457 result in increased taurocholate-independent germination. (A) Germinant sensitivity of *cspC* mutant spores encoding mutations in the flexible Arg456 and Gly457 when plated on BHIS containing increasing concentrations of taurocholate. The number of colony forming units (CFUs) produced by germinating spores is shown. The mean and standard deviations shown are based on three biological replicates performed on three independent spore purifications. Lower error bars have been omitted to improve readability. Statistical significance relative to wild type was determined using a two-way ANOVA and Tukey’s test. **** p < 0.0001, *** p < 0.001, ** p < 0.01, * p < 0.05. (B) Western blot analyses of CspC and CspB levels in G457 and R456 mutant spores. CotA serves as a loading control. The results are representative of three biological replicates performed on three independent spore preps.

To test whether bile acid-independent germination would also be observed with the R456G substitution, we measured the germination sensitivity of CspC_R456G_ and CspC_G457R_ spores on BHIS lacking germinant and with increasing amounts of taurocholate. The R456G and G457R mutant spores exhibited increased taurocholate-independent germination with a >1,000-fold increase in CFUs on BHIS alone relative to wild-type (Fig 5A). CspC_R456G_ and CspC_G457R_ spore germination steadily increased in response to increasing taurocholate and reached maximum germination at 0.01% taurocholate, whereas wild-type and *ΔcspC/*cspC spore germination reached maximum germination at 0.1% taurocholate (Fig 5A), with 0.1% taurocholate being the maximum concentration tested.

Since the G457R mutation, and the R456G substitution result in consecutive arginines or glycines, respectively, we tested whether we could revert the taurocholate-independent germination phenotype back to wild-type levels by generating a R456G-G457R double mutant. This mutation reverses the order of amino acids at residues 456 and 457 relative to the wild-type protein. Interestingly, CspC_R456G/G457R_ spores exhibited an intermediate phenotype between CspC_R456G_ and CspC_G457R_ spores when plated on media with varying levels of taurocholate (Fig 5A), with CspC_R456G-G457R_ spores forming colonies on media lacking germinant or containing 0.001% taurocholate ~3-fold less efficiently than CspC_G457R_ spores, although this difference was not statistically significant. In contrast, CspC_R456G_ spores formed colonies ~10-fold less efficiently than CspC_G457R_ spores on this media (p < 0.02). Regardless, all three mutants produced spores with wild-type amounts of CspC as determined by western blotting, suggesting that the CspC variants have enhanced signaling properties (Fig 5B).

We next questioned what effect the identity of the residue at position 457 had on spore germination. To this end, we mutated Gly457 to another small, neutral amino acid (alanine), a polar residue (glutamine), a negatively charged residue (glutamic acid), and a smaller, positively charged amino acid (lysine). CspC_G457A_ exhibited wild-type germination responses on BHIS alone and in response to different concentrations of taurocholate (S3A Fig). CspC_G457E_ and CspC_G457Q_ spores exhibited an intermediate phenotype between that of wild-type and CspC_G457R_ spores at lower taurocholate concentrations, while CspC_G457K_ spores behaved identically to CspC_G457R_ spores (S3A Fig). Western blot analysis indicated that none of these mutations affected CspC levels in mature spores (S3B Fig). Taken together, polar and charged amino acid substitutions of Gly456 (glutamic acid, lysine, and arginine) result in increased levels of taurocholate-independent germination, with positively-charged amino acids having the greatest effect.

### Mutations of the flexible residues G457 and R456 increase sensitivity to taurocholate

Since high basal levels of germination in the absence of taurocholate made it difficult to distinguish in the plate-based taurocholate titration assay if the Arg456 and Gly457 mutations increase germinant sensitivity, we assessed their germinant sensitivity using the optical density-based germination assay. CspC_R456G_ and CspC_G457R_ spores were exposed to increasing concentrations of taurocholate in the presence of BHIS, and the decrease in optical density was measured compared to wild-type spores and Δ*cspC* spores. Both CspC_R456G_ and CspC_G457R_ spores germinated more quickly at all concentrations of taurocholate tested compared to wild-type spores (S4 Fig). While the difference between wild-type and CspC_G457R_ spores was statistically significant for all taurocholate concentrations tested, CspC_R456G_ spores exhibited statistically significant increases in germination for the 0.5% (10 mM) taurocholate concentration, although it approached significance at the lowest taurocholate concentration tested (0.125%, ~2 mM).

Notably, no decrease in optical density was observed when CspC_R456G_ and CspC_G457R_ spores were incubated in BHIS alone, suggesting that no germination occurs during the 90 min time period analyzed in the optical density assay for these mutant spores in contrast with the plate-based germination assays. However, this apparent discrepancy could result from the optical density assay lacking sufficient sensitivity to detect changes in <5% of the population [43] or because the germination of these spores in the absence of bile acids occurs on a slower time scale than the 90 min analyzed (Figs 4 and 5 and S4 Fig). Thus, in addition to having increased taurocholate-independent germination (Fig 5), the R456G and G457R variants have increased sensitivity to taurocholate germinant.

### A D429K mutation also leads to taurocholate-independent germination

**Fig 6:**
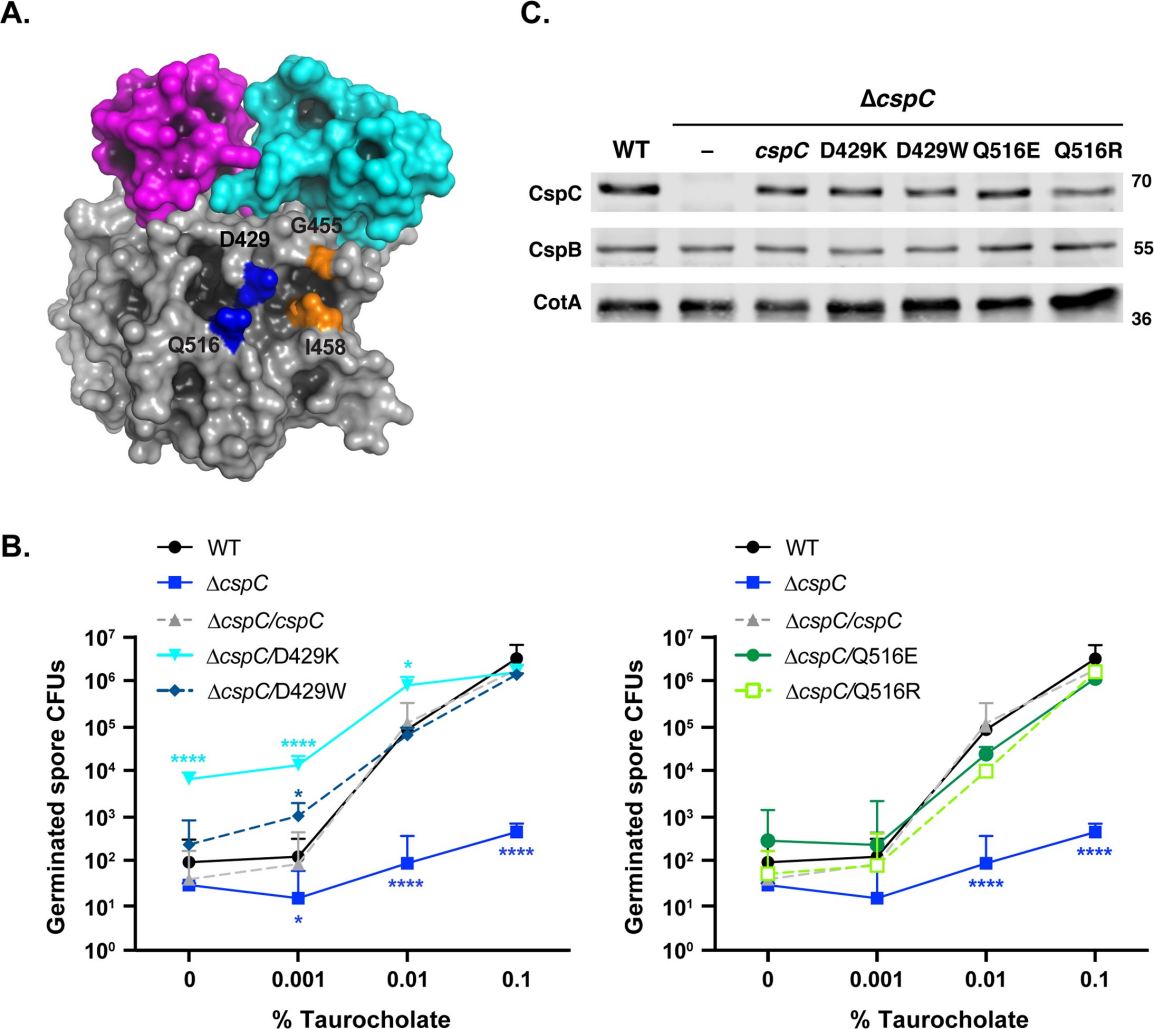
Mutation of Asp429 to Lys increases taurocholate-independent germination. (A) Space fill model of CspC highlighting the residues flanking the short loop containing R456 and G457 (orange spheres) juxtaposed to D429 and Q516 (blue spheres). A shallow cavity on the surface of the subtilase-like domain is visible between these four residues. The image is rendered to enhance cavity contrast using PyMOL (Schrödinger, Inc.) (B) Germinant sensitivity of *cspC* mutant spores encoding mutations in Asp429 and Gln516 when plated on BHIS containing increasing concentrations of taurocholate. The number of colony forming units (CFUs) that arose from germinating spores is shown in two graphs to improve readability. The mean and standard deviations shown are based on three replicates performed on three independent spore purifications. Lower error bars have been omitted to improve readability. Statistical significance relative to wild type was determined using a two-way ANOVA and Tukey’s test. **** p < 0.0001, * p < 0.05. (B) Western blot analyses of CspC and CspB levels in D429 and Q516 mutant spores. CotA serves as a loading control. The results are representative of three biological replicates performed on three independent spore preps.

The Arg456 and Gly457 residues are located on one edge of a pocket that could potentially bind small molecules, like taurocholate, so we tested if other residues in this region impact sensitivity to taurocholate. To this end, we made substitutions at residues on the other side of this pocket in aspartate 429 and glutamine 516. The substitutions either introduced a bulkier residue or reversed the charge of the native residue to potentially occlude ligand binding by this pocket (Fig 6A). Of the four substitutions tested, only CspC_D429K_ exhibited a germination profile similar to CspC_R456G_ and CspC_G457R_ spores, with CspC_D429K_ spores germinating at ~100-fold higher levels than wild type when plated on BHIS alone (Fig 6B, p < 0.0001). Although germination saturated at 0.01% taurocholate like CspC_R456G_ and CspC_G457R_ spores, the shape of its germinant sensitivity curve resembled that of wild type in that its germination on 0.001% TA was only ~2-fold higher than in the absence of TA. In contrast, CspC_R456G_ and CspC_G457R_ spore germination increased by ~8-fold, when comparing the germination levels on BHIS alone to BHIS containing 0.001% TA (Fig 5A). Consistent with these observations, CspC_D429K_ spores did not exhibit greater sensitivity to taurocholate in the optical density assay than wild-type spores (S4 Fig), in contrast with CspC_R456G_ and CspC_G457R_ spores, indicating that the *D429K* allele results in a different germinant profile than the *R456G* and *G457R* alleles. Importantly, none of the amino acid substitutions affected CspC levels in mature spores relative to wild-type (Fig 6C).

### Mutants with increased taurocholate-independent germination are more sensitive to co-germinants

Although CspC_R456G_ and CspC_G457R_ spores differed from CspC_D429K_ spores in their sensitivity to taurocholate (S4 Fig), it was unclear why all three mutant spores germinated significantly better on plates in the absence of taurocholate germinant than wild-type spores (Figs 5 and 6). Since Sorg and Sonenshein previously established that efficient *C*. *difficile* spore germination requires a second co-germinant signal [24], namely amino acids [44], we considered whether mutations in CspC could confer differential responsiveness to components within both rich and defined media. These small molecules enhance sensitivity to taurocholate without causing germination on their own [19, 20]. Recently, Kochan *et al*. determined that divalent cations, particularly calcium, also potentiate taurocholate-induced germination and that amino acid and calcium co-germinant signals can synergize to further enhance taurocholate-induced germination [45, 46].

**Fig 7:**
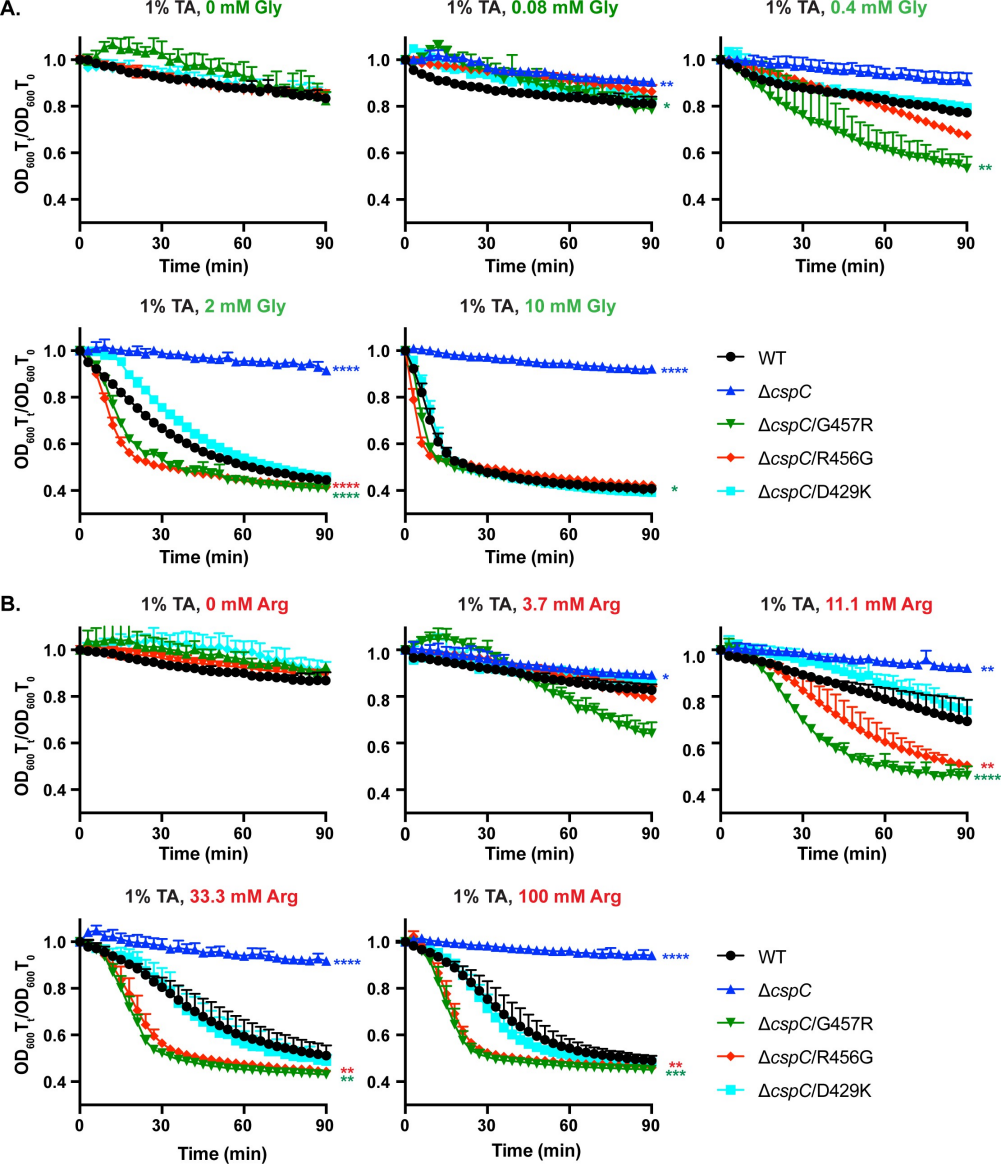
G457R and R456G mutations enhance sensitivity to amino acid co-germinants. (A) Optical density (OD_600_) analyses of spore germination over time in G457 region mutants. Purified spores from the indicated strains were incubated in PBS supplemented with 1% taurocholate and increasing concentrations of either (A) glycine or (B) arginine. The change in OD_600_ represents the OD_600_ of the sample at a given timepoint relative to its starting OD_600_ at time zero. The averages of three replicates are shown and are representative of three independent spore preps. The error bars indicate the standard deviation for each timepoint measured. Lower error bars have been omitted to improve readability. Statistical significance relative to wild type was determined using a two-way ANOVA and Tukey’s test. **** p < 0.0001, *** p < 0.001, ** p < 0.01, * p < 0.05.

Since it is currently unknown how *C*. *difficile* spores sense and respond to these co-germinants, but both amino acid and calcium co-germinants are present in the BHIS media used in our germination assays, we tested the sensitivity of CspC_R456G_, CspC_G457R_, and CspC_D429K_ spores to various co-germinants individually in the optical density assay using a PBS-based buffer rather than BHIS. We first tested the sensitivity of CspC_R456G_, CspC_G457R_, and CspC_D429K_ spores to the most potent amino acid co-germinant, glycine, and an amino acid co-germinant with a mid-range activating concentration (EC_50_), arginine [44] in the presence of constant amounts of taurocholate (1%, 19 mM). Importantly, wild-type, CspC_R456G_, CspC_G457R_, and CspC_D429K_ spores did not germinate in PBS with 1% taurocholate alone (Fig 7) or in PBS containing glycine alone (S5 Fig), just as BHIS alone did not induce germination in the optical density assay (S4 Fig). Thus, both germinant and a co-germinant must be present to detect germination in this assay.

CspC_G457R_ spores germinated in lower concentrations of glycine (0.4 mM) than wild-type, CspC_R456G_, and CspC_D429K_ spores in the presence of 1% taurocholate and germinated faster than wild-type and CspC_D429K_ spores through all glycine concentrations tested (Fig 7A, p < 0.05). At 2 mM glycine in the presence of 1% taurocholate, CspC_R456G_ spores germinated faster than wild-type and CspC_D429K_ spores (Fig 7A, p < 0.0001). CspC_G457R_ and CspC_R456G_ spores also germinated faster than wild-type and CspC_D429K_ spores at 11.1 mM arginine with 1% taurocholate (Fig 7B, p < 0.01), with CspC_G457R_ spores germinating the fastest and at a lower arginine concentration (3.7 mM, Fig 7B), although this difference was not statistically significant. Both CspC_R456G_ and CspC_G457R_ spores reached a maximum germination rate at 33.3 mM arginine with 1% taurocholate, while wild-type and CspC_D429K_ spores germination rates were still increasing at 100 mM arginine with 1% taurocholate (Fig 7B). Taken together, these data indicate that CspC_R456G_ and CspC_G457R_ spores are more sensitive to glycine and arginine co-germinants in the presence of taurocholate, while CspC_D429K_ spores respond to these amino acid co-germinants similarly to wild-type spores.

**Fig 8:**
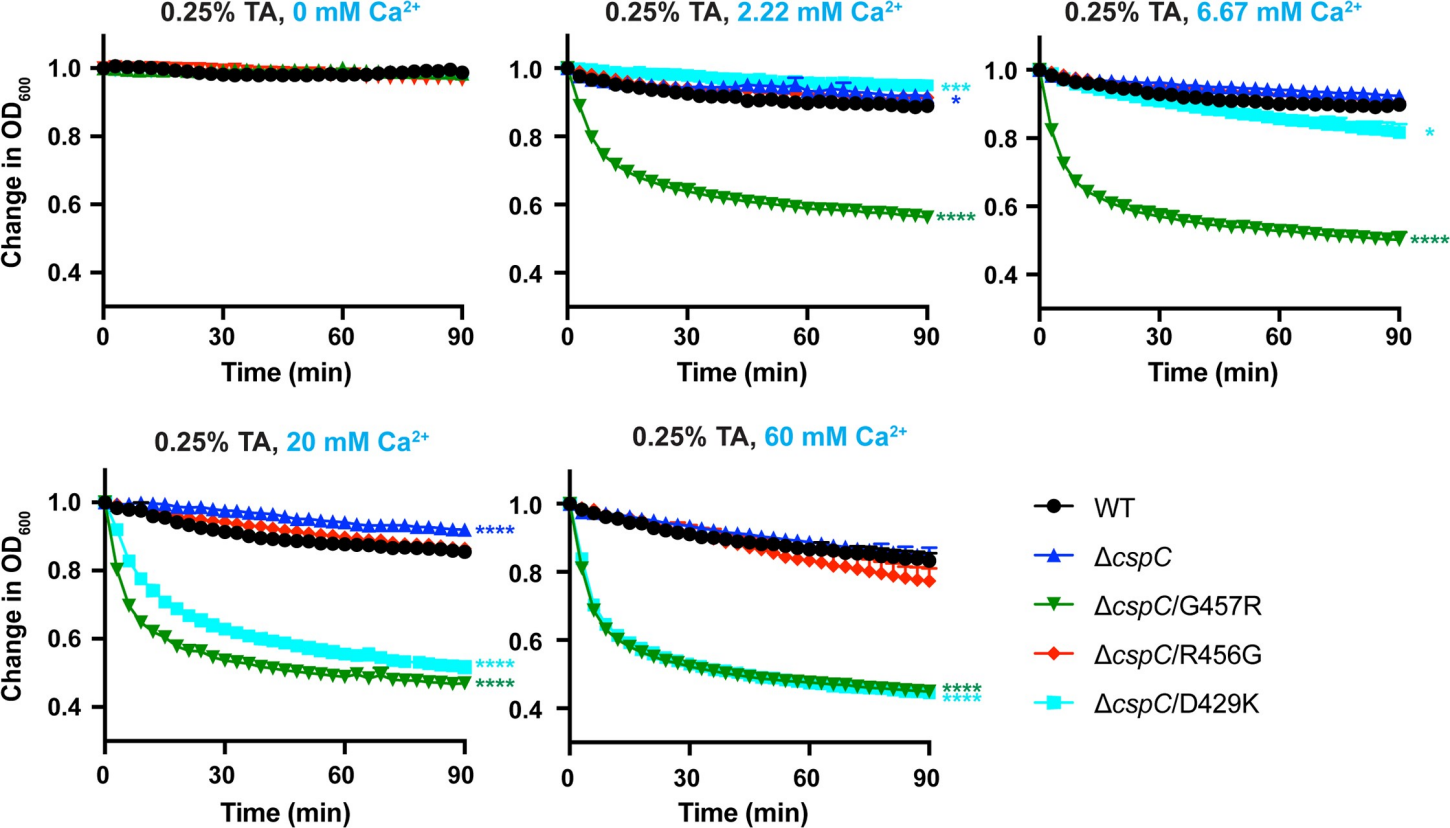
G457R and D429K mutations enhance sensitivity to the co-germinant, Ca^2+^. Optical density (OD_600_) analyses of spore germination over time in G457 region mutants. Purified spores from the indicated strains were incubated in Tris supplemented with 0.25% taurocholate and increasing concentrations of calcium. The change in OD_600_ represents the OD_600_ of the sample at a given timepoint relative to its starting OD_600_ at time zero. The averages of three replicates are shown and are representative of three independent spore preps. The error bars indicate the standard deviation for each timepoint measured. Lower error bars have been omitted to improve readability. Statistical significance relative to wild type was determined using a two-way ANOVA and Tukey’s test. **** p < 0.0001, *** p < 0.001, * p < 0.05.

We next tested the sensitivity of these CspC mutants to calcium co-germinant. In PBS buffer with CaCl_2_ added, the spores clumped together, so we used Tris buffer to test the sensitivity of the CspC mutants to calcium. We also decreased the taurocholate concentration to 0.25% to determine the sensitivity of the spores to Ca^2+^, since spores germinated too rapidly in 1% taurocholate supplemented with CaCl_2_ to accurately measure the change in optical density in a plate reader. As observed with amino acid co-germinants, the optical density of all spores tested did not change in Tris buffer containing 0.25% taurocholate and no calcium (Fig 8) or Tris buffer with 60 mM Ca^2+^ (S5 Fig). However, CspC_G457R_ spores were highly sensitive to calcium ions in the presence of taurocholate, germinating at near maximal rates at the lowest calcium concentration tested (2.22 mM CaCl_2_, Fig 8, p < 0.0001). CspC_D429K_ spores were also very sensitive to calcium, with the entire population germinating in response to 20 mM Ca^2+^ (p < 0.0001). In contrast, wild-type and CspC_R456G_ spores did not germinate appreciably in response to 60 mM Ca^2+^ (Fig 8), a result that differs slightly from that of Kochan *et al*., who first identified calcium as a co-germinant [46]. However, increasing the taurocholate levels to 1% in Tris buffer resulted in similar responses as Kochan *et al*. The different germinant sensitivities observed between the two studies could result from differences in the sporulation media (Clospore broth [46] vs. 70:30 plates) and spore purification methods. Regardless, our results indicate that CspC_R456G_, CspC_G457R_, and CspC_D429K_ spores exhibit differential sensitivity to one or more classes of co-germinants. This heightened sensitivity correlates with their increased taurocholate-independent germination on plates.

## Discussion

Unlike the nutrient germinants used by most spore-forming bacteria, the nosocomial pathogen, *C*. *difficile*, uses mammalian-specific bile acids as the signal for initiating germination. *C*. *difficile* also lacks the membrane-bound germinant receptors common to almost all spore-forming bacteria [22, 47] and instead employs the soluble, Peptostreptococceae-specific CspC pseudoprotease to sense bile acid germinants [12]. By solving the X-ray crystal structure of CspC, we identified residues, particularly residues located in flexible regions, that are critical for CspC’s signaling function. Our mutational analyses surprisingly revealed that *C*. *difficile* CspC responds not only to bile acid germinant but also to two different classes of co-germinants: amino acids and calcium (Figs 7 and 8). Since the mechanism by which co-germinants are sensed by *C*. *difficile* was previously unknown, these observations provide important insight into how *C*. *difficile* spores integrate multiple environmental signals to induce germination.

In contrast with a prior report [12], we also observed that a G457R substitution in CspC does not result in spores that germinate in response to chenodeoxycholate (Fig 4 and S1 Fig). Instead, the G457R mutation allows *C*. *difficile* spores to germinate on media in the absence of added bile acids and heightens the sensitivity of spores to taurocholate, amino acids, and calcium ions. Mutations of Gly457 to charged residues potentiated bile acid-independent spore germination (S3 Fig), suggesting that charged residues may promote interactions of CspC with (co)-germinants and/or signal transduction proteins. As we discuss later, these findings raise the possibility that *C*. *difficile* CspC may not bind bile acid germinants directly.

Our structure-guided mutational analyses identified additional residues that regulate CspC function beyond Gly457, namely the conformationally flexible residues, Arg358 and Arg456. These residues all shared the property of exhibiting greater than average mobility within the CspC crystal structure. Mutation of these residues altered *C*. *difficile* CspC’s sensitivity to bile acid germinants (Fig 3 and S4 Fig), suggesting that conformational mobility is important for CspC function. This finding was somewhat surprising given that a unique hinge and clamp region in CspC provides a larger contact surface area between the prodomain and subtilase domain than in CspB (Fig 1A and S1 Table). Nevertheless, despite this larger contact surface, CspC was surprisingly more flexible than CspB in limited proteolysis analyses (Fig 2).

Mutation of the conformationally flexible residue, Arg358, in the jelly roll domain to chemically distinct residues (alanine, glutamatic acid, and leucine) resulted in hypo-sensitivity to taurocholate germinant and a dramatically reduced germination rate (Fig 3). Although Arg358 can form a salt bridge with the nearby Glu43, this interaction appears dispensable for CspC function, since an E43A mutation did not affect germinant sensitivity (Fig 3). Thus, Arg358’s ability to interact with the surrounding environment likely determines CspC’s ability to sense germinant and/or transduce the germinant signal.

Mutation of the flexible residue, Arg456, to glycine resulted in bile acid-independent spore germination (Fig 5) as well as increased sensitivity to taurocholate germinant (S4 Fig) and amino acid co-germinants similar to the G457R mutation (Fig 7), although unlike the G457R allele, the R456G allele did not enhance the sensitivity of *C*. *difficile* spores to calcium ion co-germinant. We also identified D429K as an additional mutation that confers bile acid-independent germination to *C*. *difficile* spores. Asp429 borders a shallow pocket next to the predicted positions of R456 and G457 (Fig 6); however, unlike the R456G and G457R mutations, the D429K mutation did not increase responsiveness to taurocholate or amino acids and instead markedly increased spore germination in response to the calcium ion co-germinant (Figs 7 and 8, S4 Fig). Taken together, the D429K mutation increases sensitivity to calcium co-germinant alone; the R456G mutation increases sensitivity to taurocholate germinant and amino acid co-germinants; and the G457R mutation exhibits the highest sensitivity to all three of these small molecule types (Figs 7 and 8, S4 Fig). Since the G457R mutation increases sensitivity to both classes of co-germinants and bile acids, it may adopt the “activated” conformation of CspC more readily than wild-type CspC analogous to a hair-trigger.

Interestingly, of the mutants we characterized, only those that were more sensitive to amino acid co-germinants were more sensitive to taurocholate germinant. Amino acid co-germinants were recently reported to act synergistically with divalent cation co-germinants, but neither class of co-germinants synergizes with members of the same class [45]. The authors hypothesized that amino acid co-germinants only synergize with divalent cation co-germinants because the different classes of co-germinants are sensed by different receptors or potentially even different pathways [20, 45]. Our current data do not distinguish between whether *C*. *difficile* spores use two separate receptors, the same receptor, or CspC itself, to sense amino acid and divalent cations, but it is interesting that the Gly457, Arg456, and D429 residues all line the edge of a pocket that could bind taurocholate and/or small molecules like amino acids. While Ca^2+^ is a co-factor in some subtilisin-like proteases [33, 48], we did not observe evidence for Ca^2+^ binding in the structure.

Indeed, the mechanism by which CspC senses and/or integrates the signals from these chemically distinct classes of molecules (bile acids, amino acids, and divalent cations) is unknown. CspC may bind bile acids, amino acids, and divalent cations directly or indirectly. If CspC indirectly senses these molecules, it presumably interacts with the direct receptors for bile acids and co-germinants. In this scenario, the mutations we identified would enhance CspC’s association with these receptors. Notably, while this manuscript was under review, Shrestha and Sorg reported the identification of specific mutations within the pseudoprotease CspA that allowed *C*. *difficile* spores to germinate exclusively in response to taurocholate and independent of either amino acid or calcium co-germinants [49]. This recent finding suggests that CspC and CspA could directly interact during germinant and co-germinant sensing. Alternatively, CspC could function through some combination of these models by directly binding germinant and a co-germinant receptor(s) or vice versa. A final model that has been proposed is that CspC alters the permeability of germinants to their “true” receptors in the cortex or core [20]. It is currently unknown whether bile acid germinants and Ca^2+^ co-germinants can pass through the outer spore membrane to reach the cortex where CspC and CspB are hypothesized to be located [19, 28, 38]. Thus, CspC may facilitate the transport of germinants and/or co-germinants to the cortex region.

While more work needs to be done to distinguish between these models, our findings also raise the question as to how CspC activation by taurocholate and co-germinants (directly or indirectly) leads to activation of the CspB protease. CspC could activate CspB through a direct interaction [19, 35, 38], as some subtilisin-like proteases form dimers [48, 50]. This mechanism would be consistent with the observation that allosteric interactions between pseudoenyzmes can activate their cognate enzymes [51–56]. A possible model for CspB activation by CspC is that direct or indirect binding of germinants and co-germinants induces a conformational change in CspC that allows CspC to bring CspB into an active conformation through a direct interaction. Our observation that CspC is conformationally flexible may reflect the need for CspC to adopt a different conformation in response to germinant and/or co-germinant signal(s) that then allows CspC to activate CspB. Further work will need to be done to test these hypotheses.

In addition to providing insight into the function of CspC, the mutations we have identified could help answer one of the major questions in the field regarding how sensitivity to germinant affects the ability of *C*. *difficile* to cause disease. Epidemic strains of *C*. *difficile* vary in the severity of the disease they cause and their sensitivity to bile acid germinant [57–61]. However, since *C*. *difficile* strains exhibit high genetic diversity, it has not been possible to directly correlate germinant sensitivity to disease severity. High sensitivity to bile acid germinants could increase the effective infectious dose of *C*. *difficile* and thus lead to more severe disease, or lower sensitivity to germinant could ensure that *C*. *difficile* spores germinate closer to the colon, the primary site of infection, and thus cause more severe disease [57, 60–62]. Testing the ability of our hypo- and hyper-sensitive mutants to colonize and cause disease in animal infection models could help answer this outstanding question in the field about *C*. *difficile* pathogenesis.

## Materials and Methods

### Bacterial strains and growth conditions

All *C*. *difficile* strain manipulations were performed with 630Δ*erm*Δ*cspCΔpyrE* [35] as the parental strain using *pyrE*-based allele-coupled exchange (ACE [63]), which allows for single copy complementation of the Δ*cspC* parental mutant from an ectopic locus. *C*. *difficile* strains are listed in S2 Table; they were grown on brain heart infusion media (BHIS) supplemented with L-cysteine (0.1% w/v; 8.25 mM), taurocholate (TA, 0.1% w/v; 1.9 mM), thiamphenicol (5–15 μg/mL), kanamycin (50 μg/mL), or cefoxitin (8 μg/mL), as needed. Cultures were grown at 37°C under anaerobic conditions using a gas mixture containing 85% N_2_, 5% CO_2_, and 10% H_2_.

*Escherichia coli* strains for HB101/pRK24-based conjugations and BL21(DE3)-based protein production are listed in S2 Table. *E*. *coli* strains were grown at 37°C, shaking at 225 rpm in Luria-Bertani broth (LB). The media was supplemented with chloramphenicol (20 μg/mL), ampicillin (50 μg/mL), or kanamycin (30 μg/mL) as indicated.

### *E*. *coli* strain construction

Plasmid constructs were cloned into DH5α and sequence confirmed using Genewiz. To construct the *cspC* mutant complementation constructs, flanking primers, #2189 and 2242 (S3 Table), were used in combination with internal primers encoding a given point mutation with Δ*cspBA* genomic DNA template. The resulting *cspC* complementation constructs carry 282 bp of the *cspBA* upstream region along with the Δ*cspBA* sequence and the intergenic region between *cspBA* and *cspC*. This slightly extended construct was necessary to generate wild-type levels of CspC when the constructs were expressed from the *pyrE* locus as previously described [35]. The primers encoding each CspC point mutation are provided in S3 Table, where all primers used are listed. For example, the G457R substitution was constructed using primer pair #2189 and 1365 to amplify a 5’ *cspC* complementation construct fragment encoding the G457R mutation at the 3’ end, while primer pair #1364 and 2242 were used to amplify a 3’ *cspC* complementation construct encoding the G457R mutation at the 5’ end. The individual 5’ and 3’products were cloned into pMTL-YN1C digested with NotI/XhoI by Gibson assembly. In some cases, the two PCR products were used in a PCR SOE [64] prior to using Gibson assembly to clone the *cspC* construct into pMTL-YN1C digested with NotI and XhoI. The resulting plasmids were transformed into *E*. *coli* DH5α, confirmed by sequencing, and transformed into HB101/pRK24.

To generate the recombinant protein expression constructs for producing CspC-His_6_ variants, primer pair #1128 and 1129 was used to amplify a codon-optimized version of *cspC* using pJS148 as the template (a kind gift from Joseph Sorg). The resulting PCR product was digested with NdeI and XhoI and ligated into pET22b cut with the same enzymes. The ligation mixture was used to transform DH5α. The G457R variant was cloned using a similar procedure except that primer pair #1128 and 1361 and primer pair #1360 and 1129 were used to PCR the 5’ and 3’ fragments encoding the G457R mutation. The resulting PCR products were joined together using PCR SOE and flanking primer pair #1128 and 1129.

### Protein purification for crystallography

*E*. *coli* BL21(DE3) strains 981 and 1721 (S2 Table) were used to produce codon-optimized CspC (wild-type and G457R, respectively) using the autoinduction method. Briefly, the indicated strains were struck out onto LB plates containing ampicillin and used to inoculate a 20 mL culture of LB containing 100 μg/mL ampicillin. The culture was grown for ~4 hrs after which it was used to inoculate 1:1000 of Terrific Broth (Affymetrix) supplemented with 5052 sugar mix (0.5% glycerol, 0.05% glucose, 0.1% alpha-lactose) and ampicillin for 60 hrs at 20˚C [65]. The cells were pelleted and then resuspended in lysis buffer (500 mM NaCl, 50 mM Tris [pH 7.5], 15 mM imidazole, 10% [vol/vol] glycerol), flash frozen in liquid nitrogen, thawed and then sonicated. The insoluble material was pelleted, and the soluble fraction was incubated with Ni-NTA agarose beads (5 Prime) for 3 hrs, and eluted using high-imidazole buffer (500 mM NaCl, 50 mM Tris [pH 7.5], 200 mM imidazole, 10% [vol/vol] glycerol) after nutating the sample for 5–10 min.

Four elution fractions were pooled and then buffer exchanged into gel filtration buffer (200 mM NaCl, 10 mM Tris pH 7.5, 5% [vol/vol] glycerol) using Amicon 30 kDa cut-off filters. The buffer-exchanged protein was concentrated to 20 mg/mL or less, and gel filtration chromatography was performed using a Superdex 200 (GE Healthcare) column. Fractions containing CspC-His6 were pooled, concentrated to ~30 mg/mL, and flash frozen in liquid nitrogen.

### Crystallization and structure determination

Purified protein was buffer exchanged into 25 mM Hepes pH 7.5, 100 mM NaCl, 1 mM TCEP with 10% [vol/vol] glycerol and concentrated to 22 mg/ml. Crystallization was performed via hanging drop vapor diffusion method by mixing of equivalent volumes of protein to crystallization reagent solution. Crystals grew in a broad range of conditions but suffered from severe twinning. This challenge was addressed by pre-incubation of the protein with 0.5 M guanidinium hydrochloride prior to mixing with crystallization reagent. Crystals grew with a reagent containing 0.2 M MES pH 6.5 and 2.5 M ammonium chloride equilibrating over a well containing 2M NaCl and incubated at room temperature. Cryoprotection was achieved by serial transfer from the growth condition to a final solution containing 1.0 M ammonium sulfate and 2.0 M lithium sulfate prior to cryo-cooling into liquid nitrogen.

Data were collected at 12 KeV on GM/CA beamline 23-ID-D at the Advanced Photon Source (Argonne National Laboratory) using a Pilatus3 6M detector with data processed to 1.55 Å using HKL2000 [66] (Table 1). The crystals were of space group C222(1) with a single molecule in the asymmetric unit. A molecular replacement search model was prepared from the subtilase-like domain of CspB (4I0W) [32] using a sequence alignment imported into Chainsaw [67] followed by truncation to a c-alpha only trace. A clear molecular replacement solution (TFZ of 10.3) was achieved using Phaser [68] within Phenix [32] and submitted to density modification using Solve/Resolve [69]. Clear density for both the prodomain and jelly roll domains was observed with model completion performed by iterative rounds of manual building, automated building using Autobuild and final refinement using Phenix with a final R_free_ of 18.6%. The following residues were not included in the model due to ambiguous electron density: 1, 89–92, 456–457, 506–508, 556–558.

**Table 1:** Crystallographic data collection and refinement statistics.

PDB ID Code	6MW4
Space Group	C222(1)
Cell a, b, c (Å)	88.6, 155.2, 91.7
Resolution Å	50–1.55
^1^(High resolution Å)	(1.61–1.55)
Reflections	91876
Completeness %	99.9 (99.9)
Multiplicity	6.5 (6.0)
Wilson B	18.1
I/SigmaI	24.2 (1.7)
Rmeas %	6.6 (17.8)
Rpim %	2.6 (28.8)
CC 1/2	0.996 (0.857)
Coordinate error Å	0.18
Phase error(˚)	18.1
R_work_ %	16.4
^2^R_free_ %	18.6
Rms bonds Å	0.006
Rms angles(˚)	0.806
Ramachandran	
Favored	98.7
Disallowed	0.37
Mean B Å^2^	
Protein (atoms)	27.1 (4214)
Solvent (atoms)	36.9 (375)
Sulfate (atoms)	56.4 (25)

^1^Numbers in parentheses denote high resolution.

^2^Represents 10% of reflections omitted from refinement.

### Limited proteolysis

Purified *C*. *perfringens* CspB or *C*. *difficile* CspC proteins were diluted to 15 μM in 10 mM Tris pH 7.5 buffer. The protein solution was aliquoted into 0.2 mL tubes. A 1 mg/mL solution of chymotrypsin in 1 mM HCl was serially diluted to generate 10-fold dilutions for use in the assay. The serially diluted chymotrypsin solutions were added to the aliquoted protein solutions for a final concentration of chymotrypsin ranging from 40 μg/mL to 0.0004 μg/mL. 1 mM HCl was added to protein samples as an untreated control. The protein and chymotrypsin mixture was incubated at 37˚C for 1 hr. Chymotrypsin activity was quenched by the addition of NuPAGE 4X LDS Sample Buffer (Invitrogen) and boiled at 98˚C for three minutes. 10 μL aliquots were resolved using a 15% SDS-PAGE gel and visualized by Coomassie staining.

### *C*. *difficile* strain construction

Complementation strains were constructed as previously described using CDDM to select for recombination of the complementation construct into the *pyrE* locus by restoring uracil prototrophy [41]. Two independent clones from each complementation strain were phenotypically characterized.

### Sporulation

*C*. *difficile* strains inoculated from glycerol stocks were grown overnight on BHIS plates containing taurocholate (TA, 0.1% w/v, 1.9 mM). The resulting colonies were used to inoculate liquid BHIS cultures, which were grown to early stationary phase before being back-diluted 1:50 into BHIS. When the cultures reached an OD_600_ between 0.35 and 0.75, 120 μL of this culture were spread onto 70:30 agar plates and sporulation was induced as previously described [70] for 21–24 hrs. Sporulating cells were harvested into phosphate-buffered saline (PBS), and cells were visualized by phase-contrast microscopy.

### Spore purification

After inducing sporulation on 70:30 agar plates for 2–3 days as previously described [71], the samples were harvested into ice-cold, sterile water. A sample was removed to examine the sporulation cultures using phase-contrast microscopy. The spore samples were then washed 6 times in ice-cold water and incubated overnight in water at 4˚C. The following day, the samples were pelleted and treated with DNase I (New England Biolabs) at 37°C for 30–60 minutes, and purified on a 20%/50% HistoDenz (Sigma Aldrich) gradient. The resulting spores were washed 2–3 times in water, and spore purity was assessed using phase-contrast microscopy (>95% pure). The optical density of the spore stock was measured at OD_600_, and spores were stored in water at 4°C.

### Germination assay

Germination assays were performed as previously described [72]. For each strain tested, the equivalent of 0.35 OD_600_ units, which corresponds to ~1 x 10^7^ spores, were resuspended in 100 μl of water, and 10 μL of this mixture were removed for 10-fold serial dilutions in PBS. The dilutions were plated on BHIS-TA, and colonies arising from germinated spores were enumerated at 20–24 hrs. Germination efficiencies were calculated by averaging the CFUs produced by spores for a given strain relative to the number produced by wild type spores based on analyses of spores from three independent preparations (i.e. three biological replicates). Statistical significance was determined by performing a one-way analysis of variance (ANOVA) on natural log-transformed data using Tukey’s test. The data were transformed because the use of three independent spore preparations resulted in a non-normal distribution.

Germination assays with chenodeoxycholate or taurocholate pretreatment were performed essentially as previously described [30]. Briefly, ~3 x 10^7^ spores (1.05 OD_600_ units) were resuspended in 150 μL water. 150 μL of BHIS was added to the spore suspensions. Aliquots of the spore suspensions were exposed to 1% taurocholate, 5% chenodeoxycholate, or water (untreated) and incubated at 37˚C for 20 minutes. 10 μL of this mixture was removed for 10-fold serial dilutions in PBS and the dilutions were plated on BHIS and BHIS with 0.1% taurocholate or CDDM and CDDM with 0.1% taurocholate. Colonies arising from germinated spores were enumerated at 20–24 hrs. Data presented are the averages of CFUs enumerated from three independent spore preparations. Statistical significance was determined by performing a one-way analysis of variance (ANOVA) on natural log-transformed data using Tukey’s test. The data were transformed because the use of three independent spore preparations resulted in a non-normal distribution.

### OD_600_ kinetics assay

For OD_600_ kinetics assays with chenodeoxycholate in 96-well plates, ~1.6 x 10^7^ spores (0.55 OD_600_ units) for each condition tested were resuspended in BHIS and 180 μL were aliquoted into three wells of a 96 well flat bottom tissue culture plate (Falcon) for each condition tested. The spores were exposed to 10 mM taurocholate (~0.5%), 5 mM chenodeoxycholate (~0.2%), or 50% DMSO (untreated) in a final volume of 200 μL. The OD_600_ of the samples was measured every 3 minutes in a Synergy H1 microplate reader (Biotek) at 37˚C with constant shaking between readings. The OD_600_ for each technical triplicate was averaged at each time point and the OD_600_ of a blank measurement (BHIS with 10 mM taurocholate, 5 mM chenodeoxycholate, or 50% DMSO alone) was subtracted from the OD_600_ of the appropriately treated spores at each time point. The change in OD_600_ over time was calculated as the ratio of the OD_600_ at each time point to the OD_600_ at time zero.

For OD_600_ kinetics assays with varying concentrations of taurocholate germinant, ~2.3 x 10^7^ spores (0.8 OD_600_ units) for each condition tested were resuspended in BHIS and 900 μL were aliquoted into a well of a 24 well suspension culture plate (CellStar) for each condition tested. The spores were then exposed to 2-fold dilutions of 1% taurocholate (19 mM) or water (untreated) in a total volume of 1 mL. The OD_600_ of the samples was measured every 3 minutes in a Synergy H1 microplate reader (Biotek) at 37˚C with constant shaking between readings. The change in OD_600_ over time was calculated as the ratio of the OD_600_ at each time point to the OD_600_ at time zero.

For OD_600_ kinetics assays with varying concentrations of co-germinants, ~2.3 x 10^7^ spores (0.8 OD_600_ units) for each condition tested were resuspended in either 1.5X PBS buffer or 50 mM Tris HCl pH 7.5 and aliquoted into a well of a 24-well plate for each condition tested. As spores clumped when CaCl_2_ was added to spores resuspended in 1.5X PBS, 50 mM Tris HCl pH 7.5 was used to measure OD600 kinetics in response to calcium as previously described [46]. 5-fold serial dilutions of 1 M glycine, 3-fold serial dilutions of 1 M arginine, 3-fold serial dilutions of 6 M CaCl_2_, or 1.5X PBS or 50 mM Tris HCl (untreated) were added to spores resuspended in the appropriate buffer to a final volume of 900 μL. The spores were then exposed to 1% taurocholate (19 mM) in a total volume of 1 mL and the OD_600_ of the samples was measured every 3 minutes in a Synergy H1 microplate reader (Biotek) at 37˚C with constant shaking between readings. The change in OD_600_ over time was calculated as the ratio of the OD_600_ at each time point to the OD_600_ at time zero.

All assays described above were performed at least five times on three independent spore preparations. Data shown are averages from three replicates performed on a single spore preparation that is representative of data obtained from independent spore preparations. The additional replicates performed can be found in the Supplementary materials, S6–S9 Figs.

### Western blot analysis

Samples for immunoblotting were prepared as previously described [73]. Briefly, sporulating cell pellets were resuspended in 100 μL of PBS, and 50 μL samples were removed and freeze-thawed for three cycles. The samples were resuspended in 100 μL EBB buffer (8 M urea, 2 M thiourea, 4% (w/v) SDS, 2% (v/v) β-mercaptoethanol) and boiled for 20 min, pelleted, and resuspended again. A small amount of sample buffer was added to stain samples with bromophenol blue. *C*. *difficile* spores (~1 x 10^7^) were resuspended in EBB buffer and processed as above. The samples were resolved by 7.5% (for sporulating cell analyses of CspBA and CspC) or 12% SDS-PAGE gels and transferred to Millipore Immobilon-FL PVDF membrane. The membranes were blocked in Odyssey Blocking Buffer with 0.1% (v/v) Tween 20 and probed with rabbit polyclonal anti-CspB [32], anti-CspA (a generous gift from Joe Sorg, Texax A&M University), or anti-CotA [38] antibodies and/or mouse monoclonal anti-pentaHis (ThermoScientific), anti-SleC [32], anti-CspC [30], or anti-SpoIVA antibodies [74]. The anti-CspB and anti-CspC antibodies were used at 1:2500 dilutions, the anti-SleC antibody was used at a 1:5000 dilution, and the anti-pentaHis, anti-SpoIVA, anti-CotA, and anti-CspA antibodies were used at a 1:1000 dilution. IRDye 680CW and 800CW infrared dye-conjugated secondary antibodies were used at 1:20,000 dilutions. The Odyssey LiCor CLx was used to detect secondary antibody infrared fluorescence emissions. Results shown are representative of analyses of three independent spore preps.

### Phase-contrast microscopy analyses of germinating *C*. *difficile* spores

Germination with chenodeoxycholate or taurocholate was performed as previously described above [30] by incubating spores with either water, taurocholate, or chenodeoxycholate for 20 min at 37˚C. The spores were pelleted to remove the inducers, re-suspended in PBS, mounted on glass slides, and analyzed by phase-contrast microscopy for evidence of germination (i.e. to monitor the transition from phase-bright to phase-dark spores).

## Supporting information

S1 Fig Chenodeoxycholate does not induce spore germination in G457R mutant spores. **Chenodeoxycholate does not induce germination of CspC**_**G457R**_ **spores.** (A) Phase-contrast microscopy analyses of the indicated spores after exposure to either water, 1% TA (19 mM) taurocholate (TA), or 0.5% (12 mM) chenodeoxycholate (CDC) for 20 minutes at 37˚C. After the treatment was washed from spores, the spores were mounted on glass slides. Results shown are representative of analyses performed on three biological replicates. (B) Optical density (OD_600_) analyses of spore germination over time in the indicated strains. Purified spores from the indicated strains were incubated in BHIS in the presence of either DMSO carrier, 10 mM taurocholate (~0.5%), or 5 mM chenodeoxycholate (~0.2%). The OD_600_ of the samples was monitored in a 96-well plate using a plate reader. The change in OD_600_ represents the OD_600_ of the sample at a given timepoint relative to its starting OD_600_ at time zero. The results shown are representative of analyses performed on three biological replicates. Averages of the results from three replicates experiments performed on a single spore preparation are shown. The error bars indicate the standard deviation for each timepoint measured. Lower error bars have been omitted to improve readability. No significant difference in spore germination was observed between the different strains treated with water or CDC, but TA treatment resulted in statistically significant differences relative to wild type (**** p < 0.0001).
(TIF)

S2 Fig Mutation of Gly457 to Arg allows for bile acid-independent germination. Spores from the indicated strains were pre-treated with either water (–), 1% TA (19 mM) taurocholate (TA), or 0.5% (12 mM) chenodeoxycholate (CDC) for 30 minutes at 37˚C then serially diluted in PBS and plated onto (A) BHIS or (B) *C*. *difficile* defined media (CDDM) [42] lacking germinant. Colonies formed after ~24 hr incubation at 37˚C are shown. The mean and standard deviations shown are based on three biological replicates performed on three independent spore purifications. Statistical significance relative to wild type was determined using a two-way ANOVA. **** p < 0.0001.
(TIF)

S3 Fig Mutation of Gly457 to charged residues increases taurocholate-independent germination. (A) Germinant sensitivity of G457 mutant spores plated on BHIS containing increasing concentrations of taurocholate. The number of colony forming units (CFUs) produced by germinating spores is shown. The mean and standard deviations shown are based on three biological replicates performed on three independent spore purifications. Lower error bars have been omitted to improve readability. Statistical significance relative to wild type was determined using a two-way ANOVA and Tukey’s test. **** p < 0.0001, *** p < 0.001, ** p < 0.01, * p < 0.05. (B) Western blot analyses of CspC and CspB levels in G457 mutant spores. CotA serves as a loading control. The results are representative of three biological replicates performed on three independent spore preps.
(TIF)

S4 Fig G457R and R456G mutations increase sensitivity to taurocholate in BHIS. Optical density (OD_600_) analyses of spore germination over time in G457 region mutants. Purified spores from the indicated strains were incubated in BHIS supplemented with increasing concentrations of taurocholate. The change in OD_600_ represents the OD_600_ of the sample at a given timepoint relative to its starting OD_600_ at time zero. The averages of the results from three replicates are shown and representative of three independent spore preps. The error bars indicate the standard deviation for each timepoint measured. Lower error bars have been omitted to improve readability. Statistical significance relative to wild type was determined using a two-way ANOVA and Tukey’s test. **** p < 0.0001, *** p < 0.001, ** p < 0.01.
(TIF)

S5 Fig Co-germinants alone do not induce spore germination in the optical density assay. Optical density (OD_600_) analyses of spore germination over time. Purified spores from the indicated strains were incubated either in (A) PBS supplemented with glycine, (B) PBS supplemented with arginine, or (C) Tris supplemented with calcium chloride. The change in OD_600_ represents the OD_600_ of the sample at a given timepoint relative to its starting OD_600_ at time zero. The averages of three biological replicates performed on three independent spore preps are shown. The error bars indicate the standard deviation for each timepoint measured. Lower error bars have been omitted to improve readability.
(TIF)

S6 Fig G457R and R456G mutations enhance sensitivity to glycine co-germinant. Optical density (OD_600_) analyses of spore germination over time in G457 region mutants. Purified spores from the indicated strains were incubated in PBS supplemented with 1% taurocholate and increasing concentrations of glycine. The change in OD_600_ represents the OD_600_ of the sample at a given timepoint relative to its starting OD_600_ at time zero. (A) The averages of three replicates on a second independent spore preparation are shown. The error bars indicate the standard deviation for each timepoint measured. Lower error bars have been omitted to improve readability. Statistical significance relative to wild type was determined using a two-way ANOVA and Tukey’s test. **** p < 0.0001, *** p < 0.001, * p < 0.05. (B) The germination profile of a third independent spore preparation.
(TIF)

S7 Fig G457R and R456G mutations enhance sensitivity to arginine co-germinant. (A) Optical density (OD_600_) analyses of spore germination over time in G457 region mutants. Purified spores from the indicated strains were incubated in PBS supplemented with 1% taurocholate and increasing concentrations of arginine. The change in OD_600_ represents the OD_600_ of the sample at a given timepoint relative to its starting OD_600_ at time zero. (A) The averages of three replicates on a second independent spore preparation are shown. The error bars indicate the standard deviation for each timepoint measured. Lower error bars have been omitted to improve readability. Statistical significance relative to wild type was determined using a two-way ANOVA and Tukey’s test. **** p < 0.0001, *** p < 0.001, * p < 0.05. (B) The germination profile of a third independent spore preparation.
(TIF)

S8 Fig G457R and D429K mutations enhance sensitivity to the co-germinant, Ca2+. Optical density (OD_600_) analyses of spore germination over time in G457 region mutants. Purified spores from the indicated strains were incubated in Tris supplemented with 0.25% taurocholate and increasing concentrations of calcium. The change in OD_600_ represents the OD_600_ of the sample at a given timepoint relative to its starting OD_600_ at time zero. (A) The averages of three replicates on a second independent spore preparation are shown. The error bars indicate the standard deviation for each timepoint measured. Lower error bars have been omitted to improve readability. Statistical significance relative to wild type was determined using a two-way ANOVA and Tukey’s test. **** p < 0.0001, *** p < 0.001, ** p < 0.01, * p < 0.05. (B) The germination profiles of a third independent spore preparation.
(TIF)

S9 Fig G457R and R456G mutations enhance sensitivity to taurocholate. Optical density (OD_600_) analyses of spore germination over time in G457 region mutants. Purified spores from the indicated strains were incubated in BHIS with increasing concentrations of taurocholate. The change in OD_600_ represents the OD_600_ of the sample at a given timepoint relative to its starting OD_600_ at time zero. (A) The averages of three replicates on a second independent spore preparation are shown. The error bars indicate the standard deviation for each timepoint measured. Lower error bars have been omitted to improve readability. Statistical significance relative to wild type was determined using a two-way ANOVA and Tukey’s test. **** p < 0.0001, *** p < 0.001, ** p < 0.01, * p < 0.05. (B) The germination profiles of a third independent spore pre
(TIF)

S1 Table Analysis of domain buried surface area calculated using PDBe PISA [75]. (DOCX)

S2 Table *C. difficile* and *E. coli* strains used in this study. (DOCX)

S3 Table Primers used in this study. (DOCX)
