# Supplementary figures and images for: The CspC pseudoprotease regulates germination of Clostridioides difficile spores in response to multiple environmental signals
Source: PLoS Genet. 2019 Jul 5;15(7):e1008224. doi: 10.1371/journal.pgen.1008224 (PMC6636752; doi:10.1371/journal.pgen.1008224)

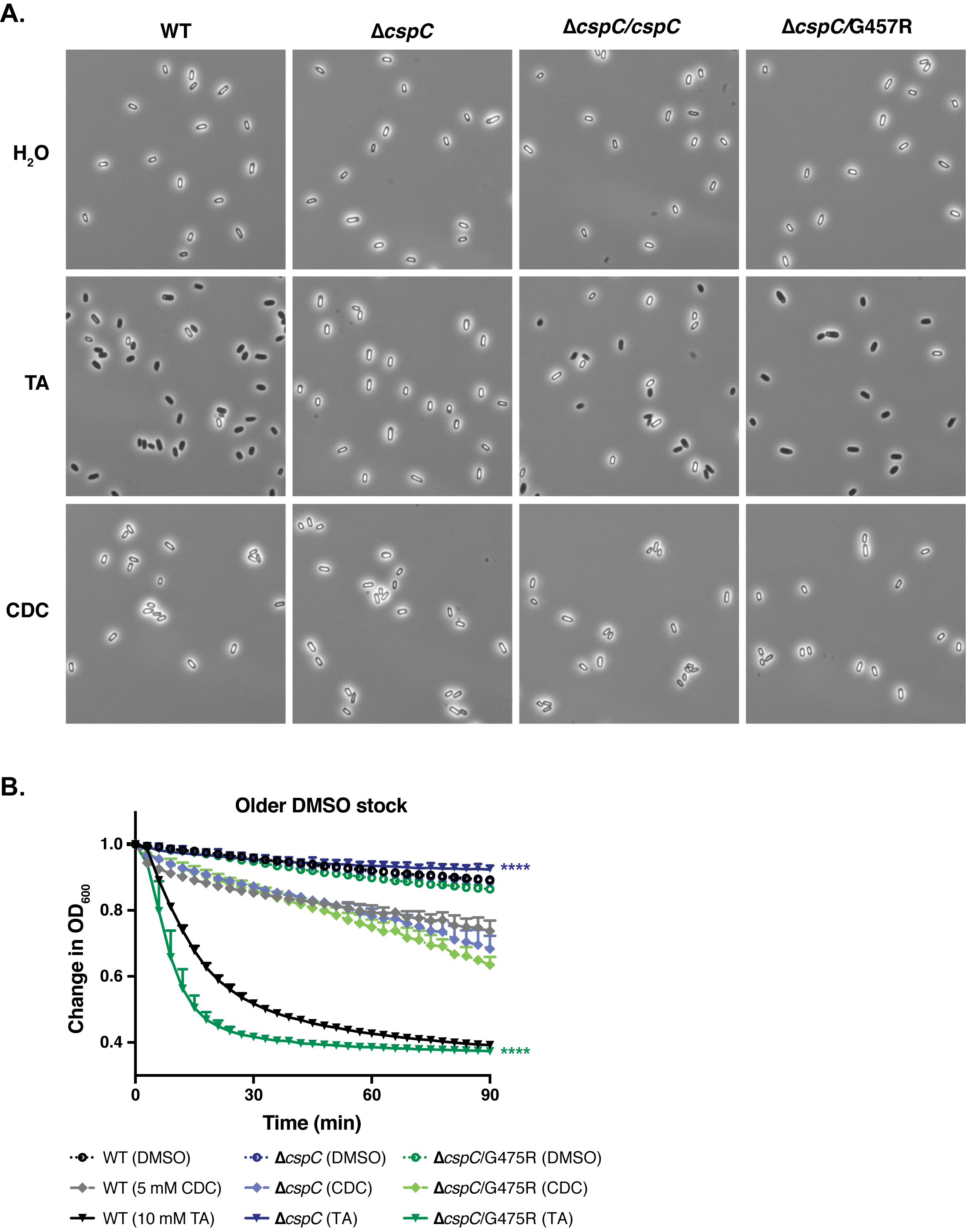

Supplement: S1 Fig — Chenodeoxycholate does not induce germination of CspCG457R spores. (A) Phase-contrast microscopy analyses of the indicated spores after exposure to either water, 1% TA (19 mM) taurocholate (TA), or 0.5% (12 mM) chenodeoxycholate (CDC) for 20 minutes at 37˚C. After the treatment was washed from spores, the spores were mounted on glass slides. Results shown are representative of analyses performed on three biological replicates. (B) Optical density (OD600) analyses of spore germination over time in the indicated strains. Purified spores from the indicated strains were incubated in BHIS in the presence of either DMSO carrier, 10 mM taurocholate (~0.5%), or 5 mM chenodeoxycholate (~0.2%). The OD600 of the samples was monitored in a 96-well plate using a plate reader. The change in OD600 represents the OD600 of the sample at a given timepoint relative to its starting OD600 at time zero. The results shown are representative of analyses performed on three biological replicates. Averages of the results from three replicates experiments performed on a single spore preparation are shown. The error bars indicate the standard deviation for each timepoint measured. Lower error bars have been omitted to improve readability. No significant difference in spore germination was observed between the different strains treated with water or CDC, but TA treatment resulted in statistically significant differences relative to wild type (**** p < 0.0001). (TIF) [file pgen.1008224.s001.tif]

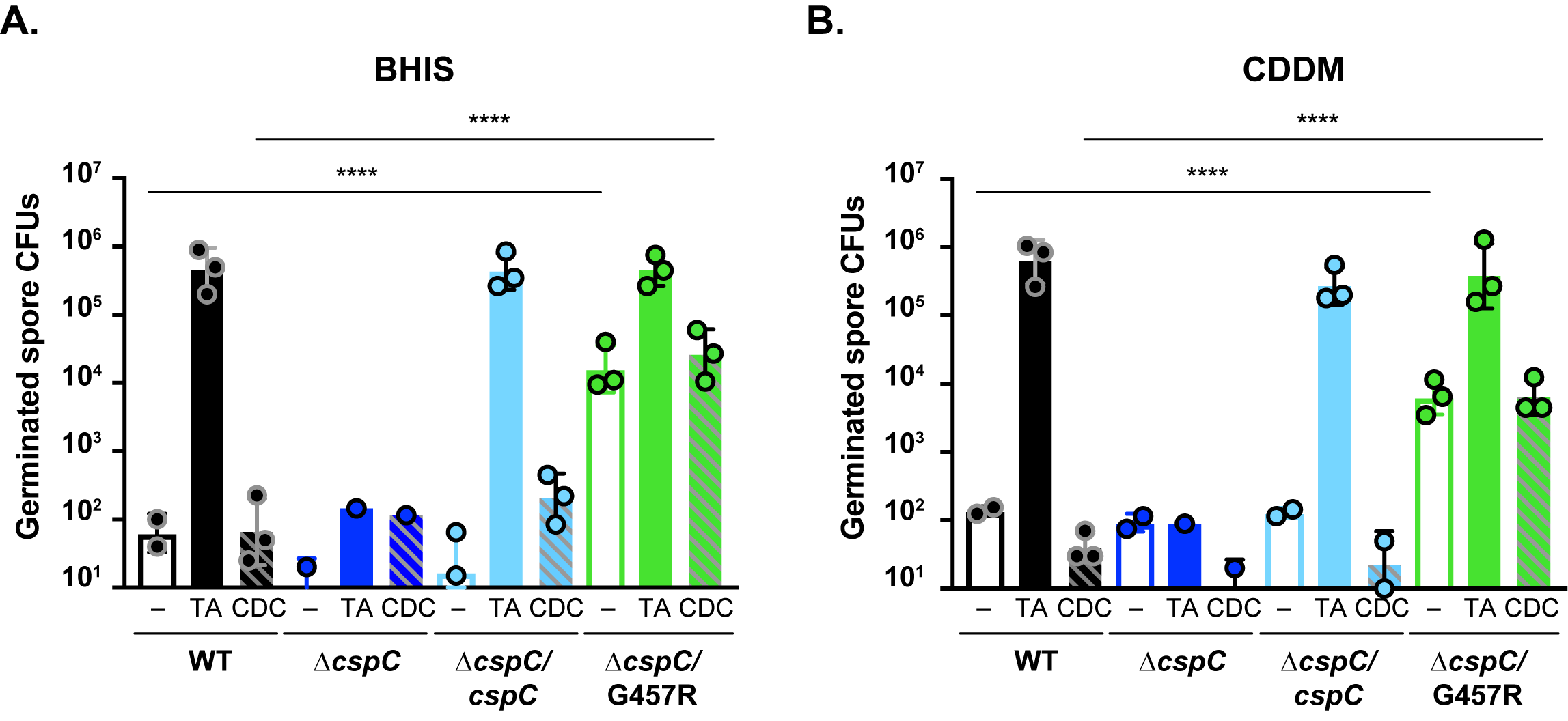

Supplement: S2 Fig — Spores from the indicated strains were pre-treated with either water (–), 1% TA (19 mM) taurocholate (TA), or 0.5% (12 mM) chenodeoxycholate (CDC) for 30 minutes at 37˚C then serially diluted in PBS and plated onto (A) BHIS or (B) C. difficile defined media (CDDM) [42] lacking germinant. Colonies formed after ~24 hr incubation at 37˚C are shown. The mean and standard deviations shown are based on three biological replicates performed on three independent spore purifications. Statistical significance relative to wild type was determined using a two-way ANOVA. **** p < 0.0001. (TIF) [file pgen.1008224.s002.tif]

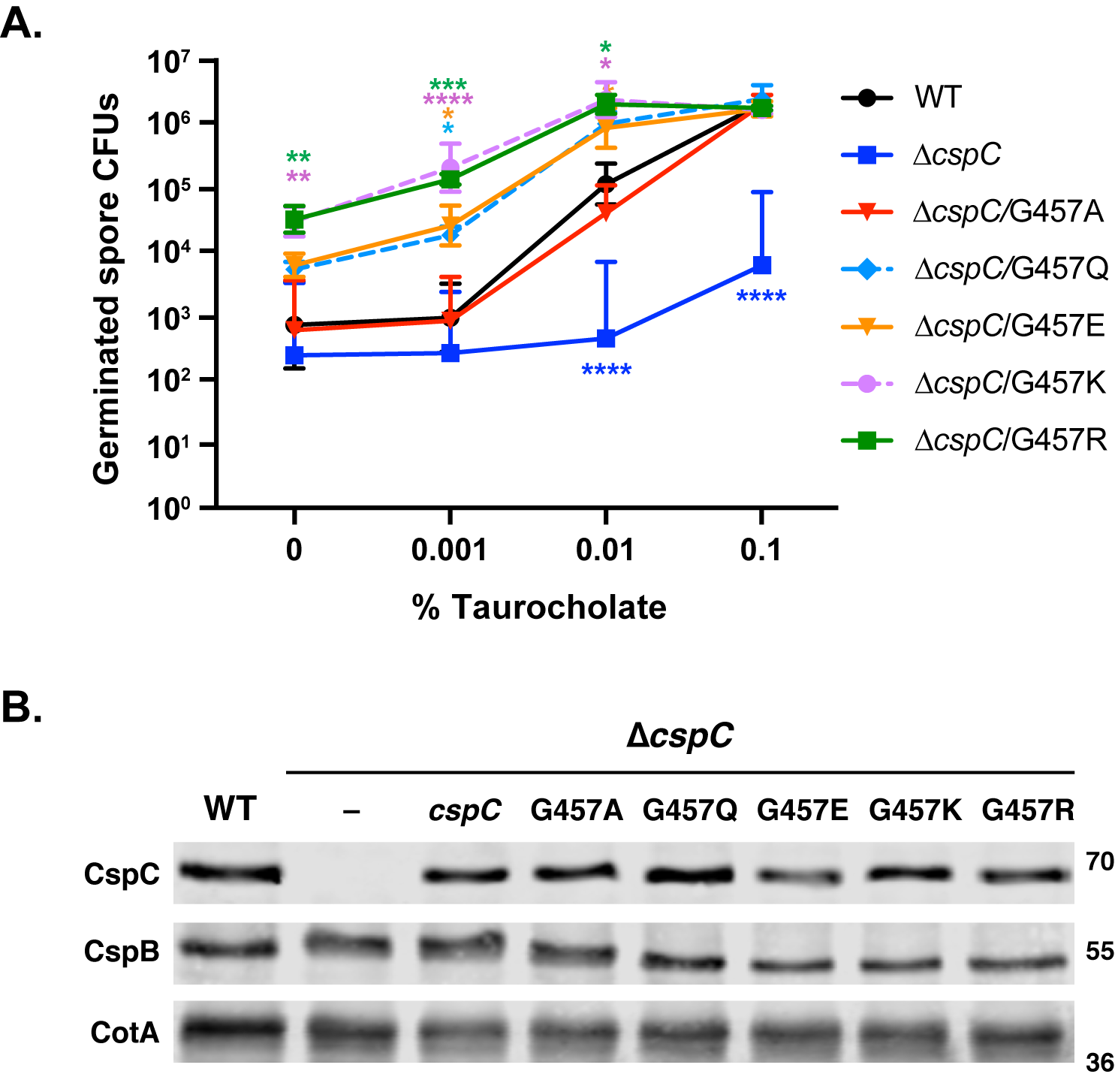

Supplement: S3 Fig — (A) Germinant sensitivity of G457 mutant spores plated on BHIS containing increasing concentrations of taurocholate. The number of colony forming units (CFUs) produced by germinating spores is shown. The mean and standard deviations shown are based on three biological replicates performed on three independent spore purifications. Lower error bars have been omitted to improve readability. Statistical significance relative to wild type was determined using a two-way ANOVA and Tukey’s test. **** p < 0.0001, *** p < 0.001, ** p < 0.01, * p < 0.05. (B) Western blot analyses of CspC and CspB levels in G457 mutant spores. CotA serves as a loading control. The results are representative of three biological replicates performed on three independent spore preps. (TIF) [file pgen.1008224.s003.tif]

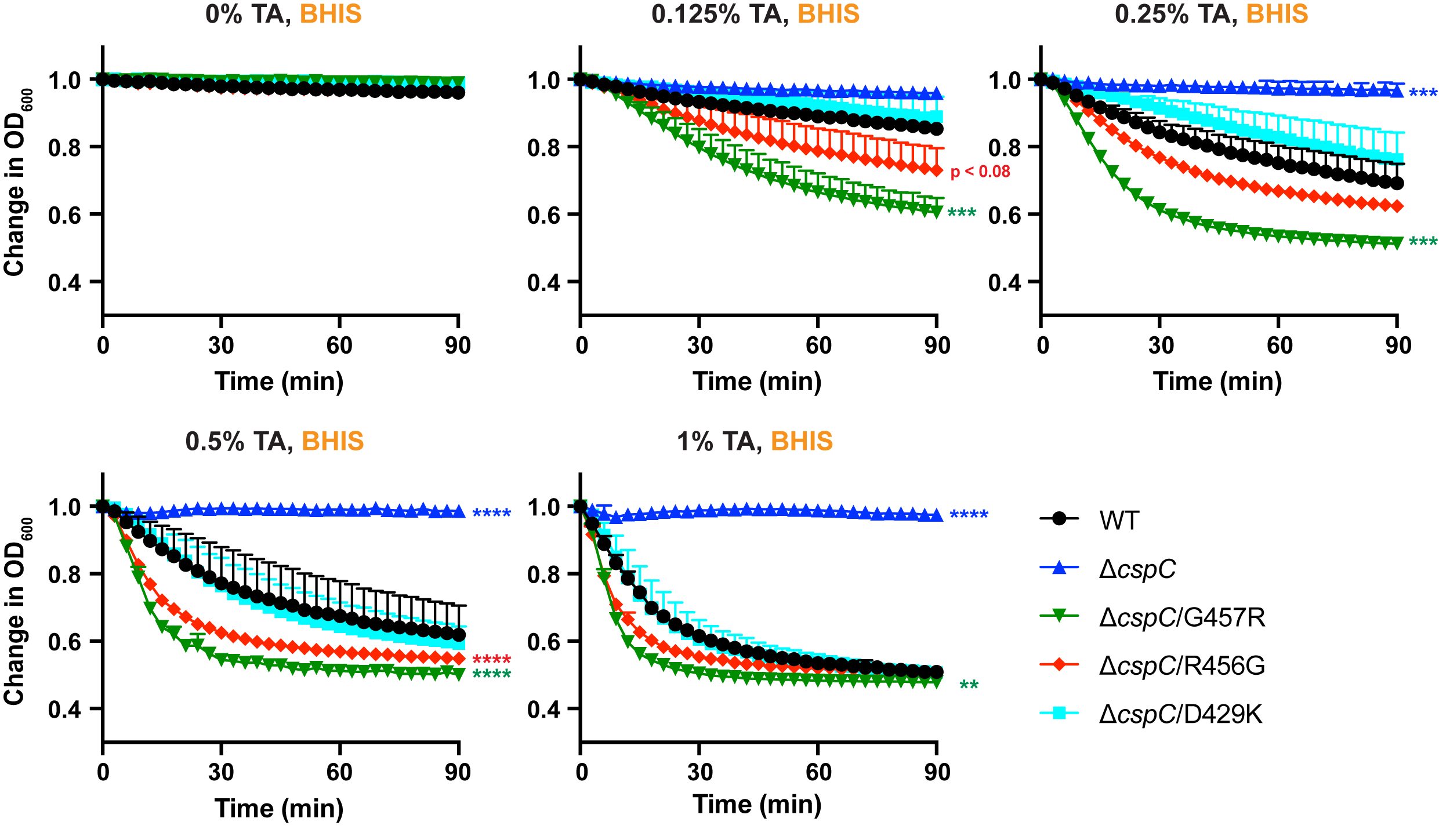

Supplement: S4 Fig — Optical density (OD600) analyses of spore germination over time in G457 region mutants. Purified spores from the indicated strains were incubated in BHIS supplemented with increasing concentrations of taurocholate. The change in OD600 represents the OD600 of the sample at a given timepoint relative to its starting OD600 at time zero. The averages of the results from three replicates are shown and representative of three independent spore preps. The error bars indicate the standard deviation for each timepoint measured. Lower error bars have been omitted to improve readability. Statistical significance relative to wild type was determined using a two-way ANOVA and Tukey’s test. **** p < 0.0001, *** p < 0.001, ** p < 0.01. (TIF) [file pgen.1008224.s004.tif]

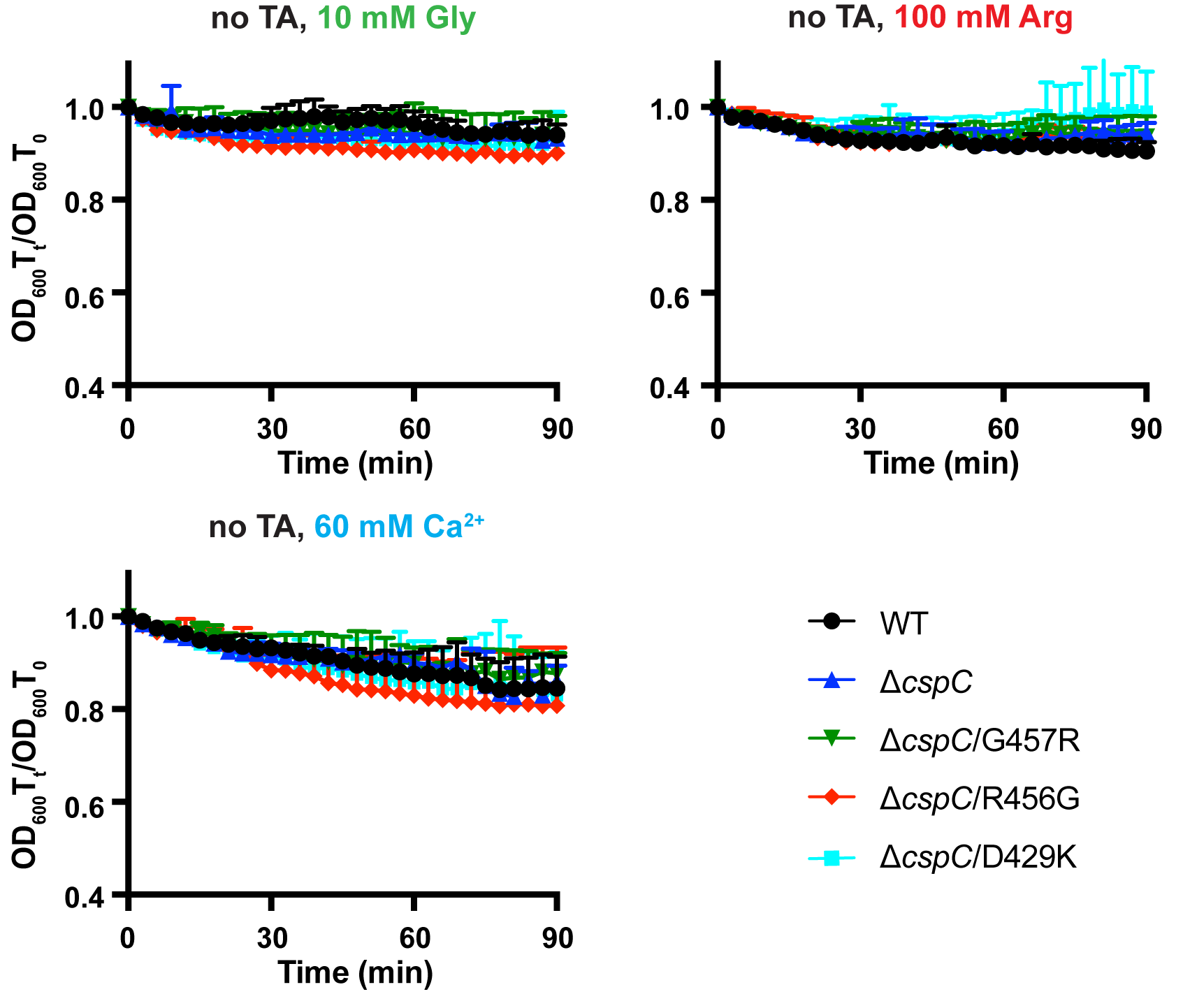

Supplement: S5 Fig — Optical density (OD600) analyses of spore germination over time. Purified spores from the indicated strains were incubated either in (A) PBS supplemented with glycine, (B) PBS supplemented with arginine, or (C) Tris supplemented with calcium chloride. The change in OD600 represents the OD600 of the sample at a given timepoint relative to its starting OD600 at time zero. The averages of three biological replicates performed on three independent spore preps are shown. The error bars indicate the standard deviation for each timepoint measured. Lower error bars have been omitted to improve readability. (TIF) [file pgen.1008224.s005.tif]

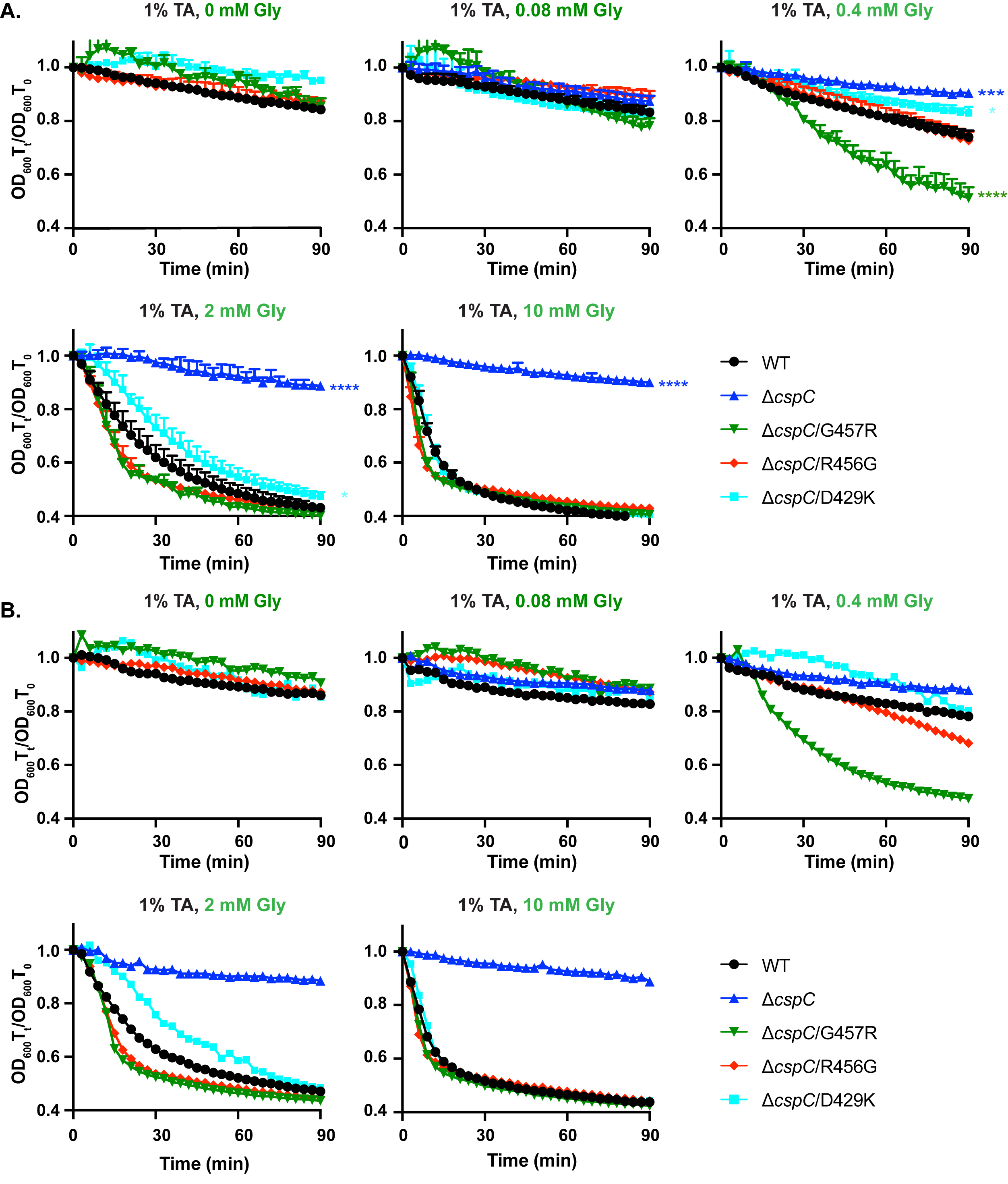

Supplement: S6 Fig — Optical density (OD600) analyses of spore germination over time in G457 region mutants. Purified spores from the indicated strains were incubated in PBS supplemented with 1% taurocholate and increasing concentrations of glycine. The change in OD600 represents the OD600 of the sample at a given timepoint relative to its starting OD600 at time zero. (A) The averages of three replicates on a second independent spore preparation are shown. The error bars indicate the standard deviation for each timepoint measured. Lower error bars have been omitted to improve readability. Statistical significance relative to wild type was determined using a two-way ANOVA and Tukey’s test. **** p < 0.0001, *** p < 0.001, * p < 0.05. (B) The germination profile of a third independent spore preparation. (TIF) [file pgen.1008224.s006.tif]

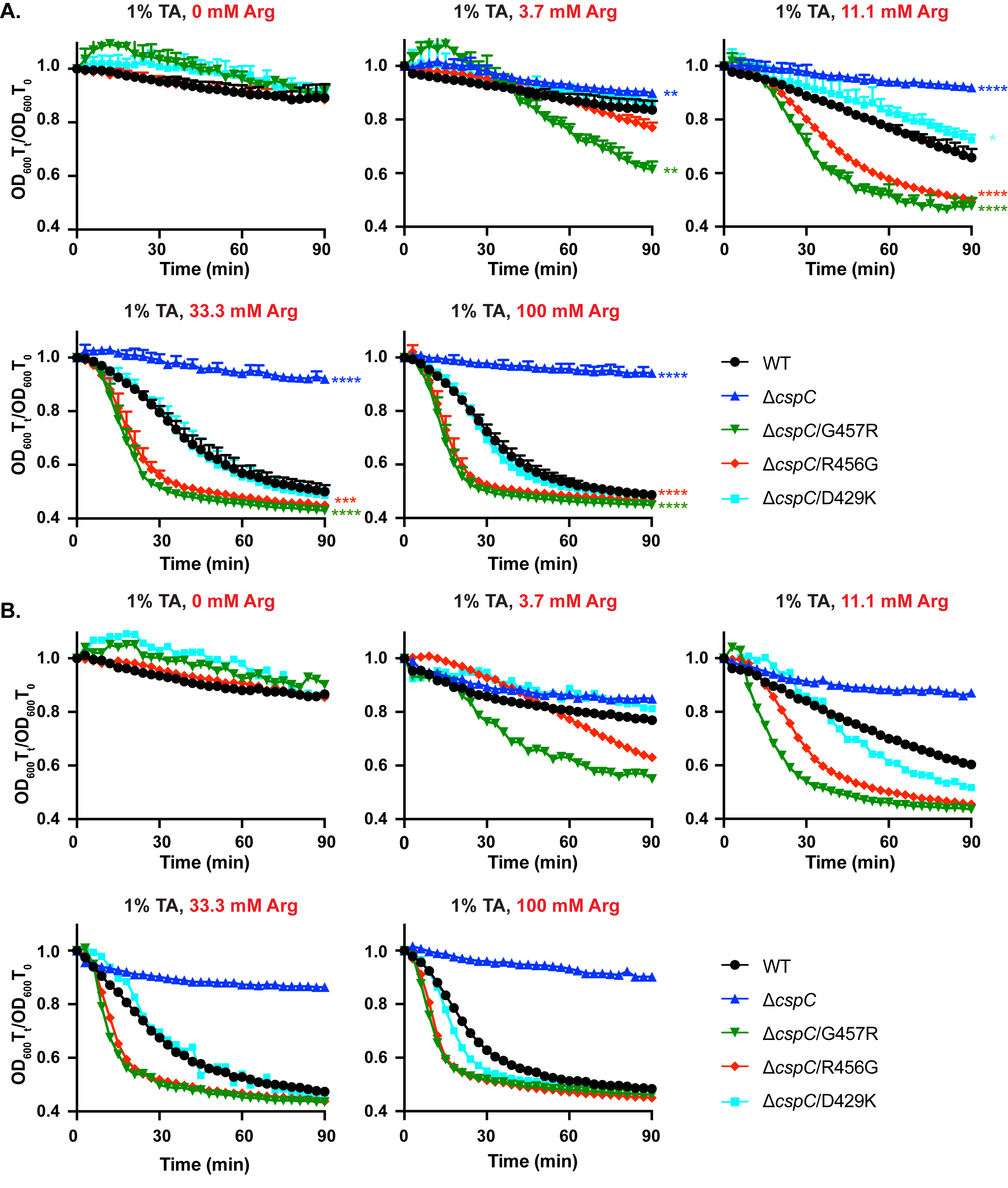

Supplement: S7 Fig — (A) Optical density (OD600) analyses of spore germination over time in G457 region mutants. Purified spores from the indicated strains were incubated in PBS supplemented with 1% taurocholate and increasing concentrations of arginine. The change in OD600 represents the OD600 of the sample at a given timepoint relative to its starting OD600 at time zero. (A) The averages of three replicates on a second independent spore preparation are shown. The error bars indicate the standard deviation for each timepoint measured. Lower error bars have been omitted to improve readability. Statistical significance relative to wild type was determined using a two-way ANOVA and Tukey’s test. **** p < 0.0001, *** p < 0.001, * p < 0.05. (B) The germination profile of a third independent spore preparation. (TIF) [file pgen.1008224.s007.tif]

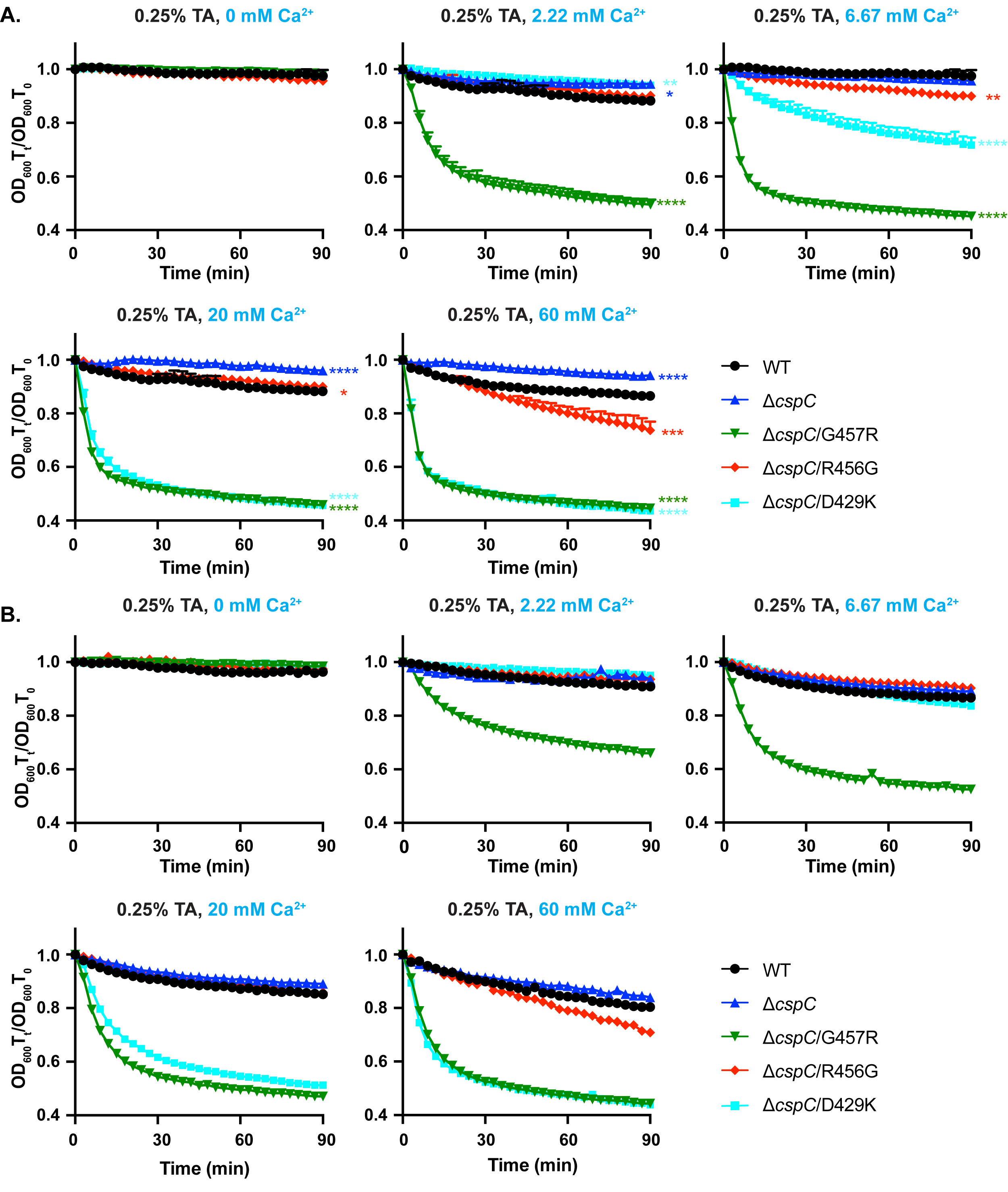

Supplement: S8 Fig — Optical density (OD600) analyses of spore germination over time in G457 region mutants. Purified spores from the indicated strains were incubated in Tris supplemented with 0.25% taurocholate and increasing concentrations of calcium. The change in OD600 represents the OD600 of the sample at a given timepoint relative to its starting OD600 at time zero. (A) The averages of three replicates on a second independent spore preparation are shown. The error bars indicate the standard deviation for each timepoint measured. Lower error bars have been omitted to improve readability. Statistical significance relative to wild type was determined using a two-way ANOVA and Tukey’s test. **** p < 0.0001, *** p < 0.001, ** p < 0.01, * p < 0.05. (B) The germination profiles of a third independent spore preparation. (TIF) [file pgen.1008224.s008.tif]

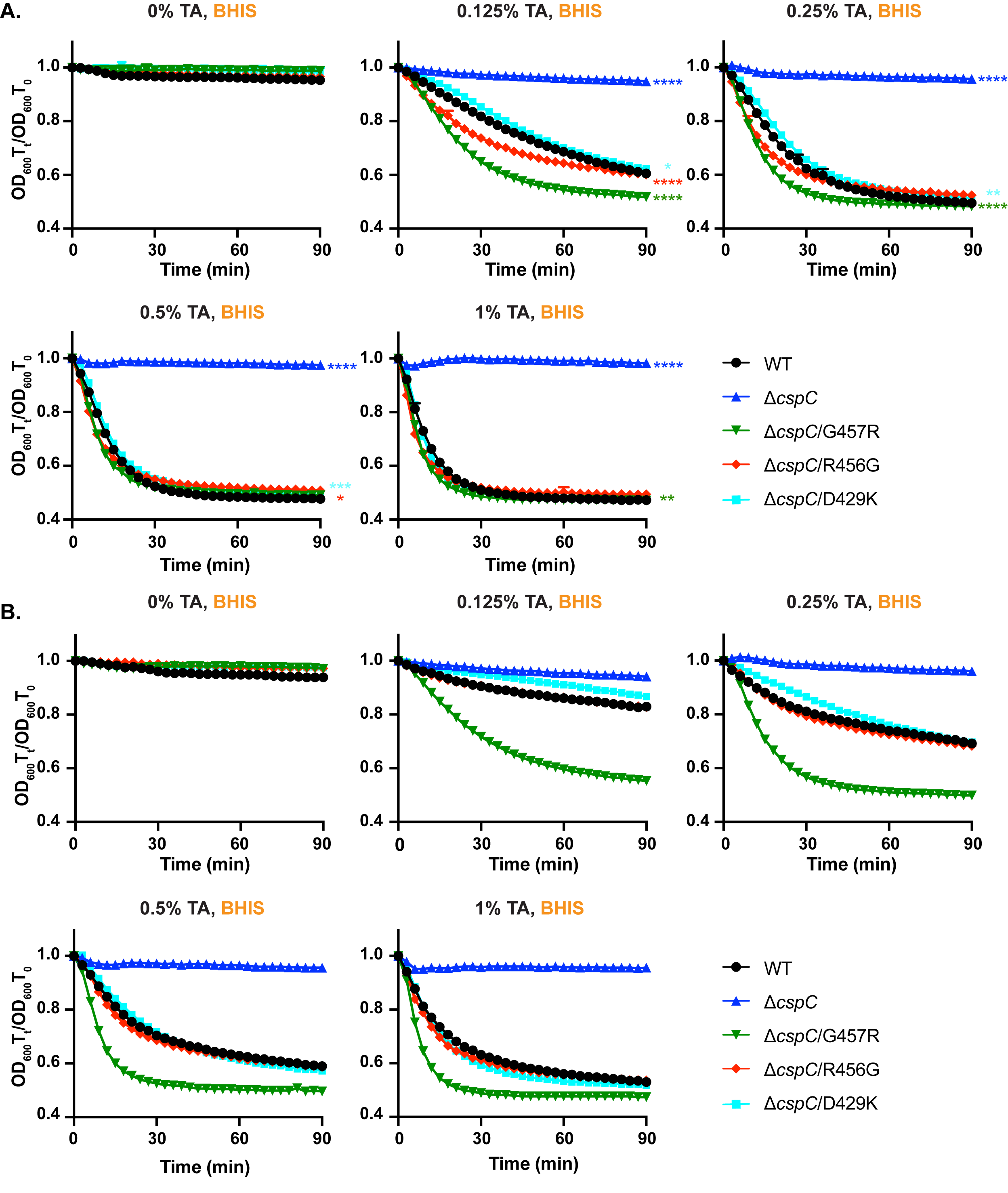

Supplement: S9 Fig — Optical density (OD600) analyses of spore germination over time in G457 region mutants. Purified spores from the indicated strains were incubated in BHIS with increasing concentrations of taurocholate. The change in OD600 represents the OD600 of the sample at a given timepoint relative to its starting OD600 at time zero. (A) The averages of three replicates on a second independent spore preparation are shown. The error bars indicate the standard deviation for each timepoint measured. Lower error bars have been omitted to improve readability. Statistical significance relative to wild type was determined using a two-way ANOVA and Tukey’s test. **** p < 0.0001, *** p < 0.001, ** p < 0.01, * p < 0.05. (B) The germination profiles of a third independent spore pre (TIF) [file pgen.1008224.s009.tif]
