# Supplementary material for: The CspC pseudoprotease regulates germination of Clostridioides difficile spores in response to multiple environmental signals
Source: PLoS Genet. 2019 Jul 5;15(7):e1008224. doi: 10.1371/journal.pgen.1008224 (PMC6636752; doi:10.1371/journal.pgen.1008224)
Supplement: S1 Table — (DOCX) [file pgen.1008224.s010.docx]

**S1 Table. Analysis of domain buried surface area calculated using PDBe PISA** [1].

|  | CspC |  |  | CspB |  |  |
| --- | --- | --- | --- | --- | --- | --- |
|  | Pro:Subt | Pro:Jelly | Subt:Jelly | Pro:Subt | Pro:Jelly | Subt:Jelly |
| Area Å^2^ | 1569 | 479 | 1151 | 1216 | 285 | 766 |
| ΔG kcal/mol | -23.2 | -2.7 | -9.6 | -15.1 | -4.9 | -0.8 |
| Hydrogen Bonds | 29 | 4 | 21 | 21 | 1 | 8 |
| Salt Bridges | 0 | 2  E43:R358  E57:R374 |  | 3  E35:R231  E59:R231  K91:D257 | 0 | 1  R462:D406 |
