# Supplementary material for: The CspC pseudoprotease regulates germination of Clostridioides difficile spores in response to multiple environmental signals
Source: PLoS Genet. 2019 Jul 5;15(7):e1008224. doi: 10.1371/journal.pgen.1008224 (PMC6636752; doi:10.1371/journal.pgen.1008224)
Supplement: S2 Table — (DOCX) [file pgen.1008224.s011.docx]

**S2 Table. *C. difficile* and *E. coli* strains used in this study.**

| **Strain#** | **Strain name** | **Relevant genotype or features** | **Source/reference** | |
| --- | --- | --- | --- | --- |
| ***C. difficile* strains** | | | |  |
| 799 | 630∆*erm*∆*pyrE*∆*cspC* | 630∆*erm*∆*pyrE* with *cspC* deleted | | [3] |
| 831 | 630∆*erm*∆*cspC*/*cspC* | 630∆*erm*∆*cspC* with *cspC* in the *pyrE* locus | | [3] |
| 846 | 630∆*erm*-p | *erm*-sensitive derivate of 630 with *pyrE* restored | | [2] |
| 1238 | 630∆*erm*∆*cspC-*p | 630∆*erm*∆*cspC* with *pyrE* restored | | [3] |
| 1766 | 630∆*erm*∆*cspC*/*cspC*_G457R_ | 630∆*erm*∆*cspC* with *cspC*_G457R_ in the *pyrE* locus | | This study |
| 1790 | 630∆*erm*∆*cspC*/*cspC*_E43A_ | 630∆*erm*∆*cspC* with *cspC*_E43A_ in the *pyrE* locus | | This study |
| 1833 | 630∆*erm*∆*cspC*/*cspC*_R358E_ | 630∆*erm*∆*cspC* with *cspC*_R358E_ in the *pyrE* locus | | This study |
| 1835 | 630∆*erm*∆*cspC*/*cspC*_R374A_ | 630∆*erm*∆*cspC* with *cspC*_R374A_ in the *pyrE* locus | | This study |
| 1841 | 630∆*erm*∆*cspC*/*cspC*_E57A_ | 630∆*erm*∆*cspC* with *cspC*_E57A_ in the *pyrE* locus | | This study |
| 1901 | 630∆*erm*∆*cspC*/*cspC*_D429W_ | 630∆*erm*∆*cspC* with *cspC*_D429W_ in the *pyrE* locus | | This study |
| 1904 | 630∆*erm*∆*cspC*/*cspC*_R358A_ | 630∆*erm*∆*cspC* with *cspC*_R358A_ in the *pyrE* locus | | This study |
| 1920 | 630∆*erm*∆*cspC*/*cspC*_D429K_ | 630∆*erm*∆*cspC* with *cspC*_D429K_ in the *pyrE* locus | | This study |
| 1953 | 630∆*erm*∆*cspC*/*cspC*_R358L_ | 630∆*erm*∆*cspC* with *cspC*_R358L_ in the *pyrE* locus | | This study |
| 1971 | 630∆*erm*∆*cspC*/*cspC*_R456G_ | 630∆*erm*∆*cspC* with *cspC*_R456G_ in the *pyrE* locus | | This study |
| 1974 | 630∆*erm*∆*cspC*/*cspC*_R456G/G457R_ | 630∆*erm*∆*cspC* with *cspC*_R456G/G457R_ in the *pyrE* locus | | This study |
| 2005 | 630∆*erm*∆*cspC*/*cspC*_Q516E_ | 630∆*erm*∆*cspC* with *cspC*_Q516E_ in the *pyrE* locus | | This study |
| 2008 | 630∆*erm*∆*cspC*/*cspC*_Q516R_ | 630∆*erm*∆*cspC* with *cspC*_Q516R_ in the *pyrE* locus | | This study |
| 2132 | 630∆*erm*∆*cspC*/*cspC*_G457Q_ | 630∆*erm*∆*cspC* with *cspC*_G457Q_ in the *pyrE* locus | | This study |
| 2134 | 630∆*erm*∆*cspC*/*cspC*_G457A_ | 630∆*erm*∆*cspC* with *cspC*_G457A_ in the *pyrE* locus | | This study |
| 2137 | 630∆*erm*∆*cspC*/*cspC*_G457E_ | 630∆*erm*∆*cspC* with *cspC*_G457E_ in the *pyrE* locus | | This study |
| 2140 | 630∆*erm*∆*cspC*/*cspC*_G457K_ | 630∆*erm*∆*cspC* with *cspC*_G457K_ in the *pyrE* locus | | This study |
| ***E. coli* strains** | |  | |  |
| **Strain#** | **Strain Background** | **Plasmid carried** | |  |
| 41 | DH5α | F– Φ80*lacZ*ΔM15 Δ(*lacZYA-argF*) U169 *recA1 endA1 hsdR17* (rK^–^, mK^+^) *phoA supE44* λ– *thi-1 gyrA96 relA1* | | D. Cameron |
| 531 | HB101/pRK24 | F- *mcrB mrr hsdS20*(rB^–^mB^–^) *recA13 leuB6 ara-13 proA2 lavYI galK2 xyl-6 mtl-1 rpsL20* carrying pRK24 | | C. Ellermeier |
| 981 | BL21(DE3) | pET22b *cspC-*His_6_ codon-optimized | | This study |
| 1721 | BL21(DE3) | pET22b *cspC*_G457R_*-*His_6_ codon-optimized | | This study |
| 2017 | HB101 | pMTL-YN1C ∆*cspBA*-*cspC*_G457R_ | | This study |
| 2028 | HB101 | pMTL-YN1C ∆*cspBA*-*cspC*_R458A_ | | This study |
| 2029 | HB101 | pMTL-YN1C ∆*cspBA*-*cspC*_E43A_ | | This study |
| 2065 | HB101 | pMTL-YN1C ∆*cspBA*-*cspC*_E57A_ | | This study |
| 2106 | HB101 | pMTL-YN1C ∆*cspBA*-*cspC*_D429K_ | | This study |
| 2107 | HB101 | pMTL-YN1C ∆*cspBA*-*cspC*_D429W_ | | This study |
| 2113 | HB101 | pMTL-YN1C ∆*cspBA*-*cspC*_R358L_ | | This study |
| 2114 | HB101 | pMTL-YN1C ∆*cspBA*-*cspC*_R456G_ | | This study |
| 2115 | HB101 | pMTL-YN1C ∆*cspBA*-*cspC*_R456G/G457R_ | | This study |
| 2120 | HB101 | pMTL-YN1C ∆*cspBA*-*cspC*_Q516E_ | | This study |
| 2121 | HB101 | pMTL-YN1C ∆*cspBA*-*cspC*_Q516R_ | | This study |
| 2182 | HB101 | pMTL-YN1C ∆*cspBA*-*cspC*_G457A_ | | This study |
| 2183 | HB101 | pMTL-YN1C ∆*cspBA*-*cspC*_G457K_ | | This study |
| 2184 | HB101 | pMTL-YN1C ∆*cspBA*-*cspC*_G457E_ | | This study |
| 2185 | HB101 | pMTL-YN1C ∆*cspBA*-*cspC*_G457Q_ | | This study |
| 2279 | HB101 | pMTL-YN1C ∆*cspBA-cspC*_R358E_ | | This study |
| 2280 | HB101 | pMTL-YN1C ∆*cspBA-cspC*_R374A_ | | This study |

| **Plasmids** |
| --- |
|  |

| **Plasmids** | **Relevant features** | **Source/reference** |
| --- | --- | --- |
| pMTL-YN1C | Unstable plasmid for restoring *pyrE* locus in 630∆*ermpyrE* | [4] |
| pET22b | Expression vector for IPTG-inducible production of C-terminally His_6_-tagged proteins | Novagen |
