# Supplementary material for: The CspC pseudoprotease regulates germination of Clostridioides difficile spores in response to multiple environmental signals
Source: PLoS Genet. 2019 Jul 5;15(7):e1008224. doi: 10.1371/journal.pgen.1008224 (PMC6636752; doi:10.1371/journal.pgen.1008224)
Supplement: S3 Table — (DOCX) [file pgen.1008224.s012.docx]

**Supplementary Table S3. Primers used in this study.**

| **Primer** | **Name** | **Sequence** |
| --- | --- | --- |
| 1128 | 5' NdeI *cspC* codon optimized from pJS148 | AAAAAACATATGGAGAAAAGCTACTGCATTATTTATC^a^ |
| 1129 | 3' XhoI *cspC* no stop codon optimized from pJS148 | AAAAAACTCGAGAAGGGTGTTGGCAATCTG |
| 1360 | 5' *cspC* G457R *cspC* CO SOE | GGCCCCATTCGTGGTCGCAGGATCAAACCGGATATCGTTGCTAG |
| 1361 | 3' *cspC* G457R *cspC* CO rev oes | CTAGCAACGATATCCGGTTTGATCCTGCGACCACGAATGGGGCC |
| 1364 | 5' *cspC* G457R diff SOE | CGAAAGGACCAATTAGAGGAAGAAGAATTAAGCCAGATATAGTAGCTTCAG |
| 1365 | 3' *cspC* G457R diff rev oes | CTGAAGCTACTATATCTGGCTTAATTCTTCTTCCTCTAATTGGTCCTTTCG |
| 2189 | 5' NotI *cspBA* promoter gibson | AATTAGGGATGTAATAAGCGGCCGCTTCAATTAATTATTGGTATCAAACT |
| 2242 | 3' XhoI *cspC* Gibson | CAAGCTTGCATGTCTGCAGGCCTCGAGCTATAGAGTATTTGCTATCTGTTGAATCG |
| 2311 | 5' NdeI pET22b *cspC* CO Gibson | GTTTAACTTTAAGAAGGAGATATACATATGGAGAAAAGCTACTGCATTATTTATCAG |
| 2312 | 3' XhoI pET22b *cspC* CO gibson | GTGGTGGTGGTGGTGGTGCTCGAGAAGGGTGTTGGCAATCTGCTGAATG |
| 2514 | 5' *cspC*(R358A) SOE | GAAAATACTACTTATGCAATGGCATTTATTTATCCATATATTACCTC |
| 2515 | 3' *cspC*(R358A) rev EOS | GAGGTAATATATGGATAAATAAATGCCATTGCATAAGTAGTATTTTC |
| 2516 | 5' *cspC*(E43A) SOE | GTAATATATGTACCTTTAGATTTTGATGCAACTATTTTAAATAATATAATACAAG |
| 2517 | 3' *cspC*(E43A) rev EOS | CTTGTATTATATTATTTAAAATAGTTGCATCAAAATCTAAAGGTACATATATTAC |
| 2563 | 5' E57A *cspC* SOE | ATAATACAAGTTGCTTGGTGGGAAGCATCTGAACCAATGAGCAGTCTAATTG |
| 2564 | 3' E57A *cspC* rev eos | CAATTAGACTGCTCATTGGTTCAGATGCTTCCCACCAAGCAACTTGTATTAT |
| 2565 | 5' R358E *cspC* SOE | GAAAATACTACTTATGCAATGGAATTTATTTATCCATATATTACCTC |
| 2566 | 3' R358E *cspC* rev eos | GAGGTAATATATGGATAAATAAATTCCATTGCATAAGTAGTATTTTC |
| 2567 | 5' R374A *cspC* SOE | CTCTGGAAAAGAAAATTTGGAGATAGCATTAAGAGATATAAAACCTGGAGTATG |
| 2568 | 3' R374A *cspC* rev eos | CATACTCCAGGTTTTATATCTCTTAATGCTATCTCCAAATTTTCTTTTCCAGAG |
| 2609 | 5' D429K *cspC* SOE | CTATAACTATGTATGCTGCTAGTAAAGATGTAATTACAGTTGGTAC |
| 2610 | 3' D429K *cspC* rev eos | GTACCAACTGTAATTACATCTTTACTAGCAGCATACATAGTTATAG |
| 2611 | 5' D429W *cspC* SOE | CTATAACTATGTATGCTGCTAGTTGGGATGTAATTACAGTTGGTAC |
| 2612 | 3' D429W *cspC* rev eos | GTACCAACTGTAATTACATCCCAACTAGCAGCATACATAGTTATAG |
| 2621 | 5' R358L *cspC* SOE | GAAAATACTACTTATGCAATGCTATTTATTTATCCATATATTACCTC |
| 2622 | 3' R358L *cspC* rev eos | GAGGTAATATATGGATAAATAAATAGCATTGCATAAGTAGTATTTTC |
| 2633 | 5' *cspC* R456G SOE | CTTCGAAAGGACCAATTAGAGGAGGAGGAATTAAGCCAGATATAGTAG |
| 2634 | 3' *cspC* R456G rev EOS | CTACTATATCTGGCTTAATTCCTCCTCCTCTAATTGGTCCTTTCGAAG |
| 2635 | 5' *cspC* R456G/G457R SOE | GTTCTTCGAAAGGACCAATTAGAGGAGGAAGAATTAAGCCAGATATAGTAGCTTCAG |
| 2636 | 3' *cspC* R456G/G457R rev EOS | CTGAAGCTACTATATCTGGCTTAATTCTTCCTCCTCTAATTGGTCCTTTCGAAGAAC |
| 2645 | 5' *cspC* Q516R SOE | GAGATTATCATTGTTTACACGAGTTTTAAAAACATACTTAATATTG |
| 2646 | 3' *cspC* Q516R rev EOS | CAATATTAAGTATGTTTTTAAAACTCGTGTAAACAATGATAATCTC |
| 2647 | 5' *cspC* Q516E SOE | GAGATTATCATTGTTTACAGAAGTTTTAAAAACATACTTAATATTG |
| 2723 | 5' *cspC* G457A SOE | CGAAAGGACCAATTAGAGGAAGAGCAATTAAGCCAGATATAGTAGCTTCAG |
| 2724 | 3' *cspC* G457A EOS | CTGAAGCTACTATATCTGGCTTAATTCTTCTTCCTCTAATTGGTCCTTTCG |
| 2725 | 5' *cspC* G457K SOE | CGAAAGGACCAATTAGAGGAAGAAAAATTAAGCCAGATATAGTAGCTTCAG |
| 2726 | 3' *cspC* G457K EOS | CTGAAGCTACTATATCTGGCTTAATTTTTCTTCCTCTAATTGGTCCTTTCG |
| 2727 | 5' *cspC* G457E SOE | CGAAAGGACCAATTAGAGGAAGAGAAATTAAGCCAGATATAGTAGCTTCAG |
| 2728 | 3' *cspC* G457E EOS | CTGAAGCTACTATATCTGGCTTAATTTCTCTTCCTCTAATTGGTCCTTTCG |
| 2729 | 5' *cspC* G457Q SOE | CGAAAGGACCAATTAGAGGAAGACAAATTAAGCCAGATATAGTAGCTTCAG |
| 2730 | 3' *cspC* G457Q EOS | CTGAAGCTACTATATCTGGCTTAATTTGTCTTCCTCTAATTGGTCCTTTCG |

^a^Restriction sites are underlined
